# Public target interventions to reduce the inappropriate use of medicines or medical procedures: a systematic review

**DOI:** 10.1186/s13012-020-01018-7

**Published:** 2020-10-20

**Authors:** Leesa Lin, Prima Alam, Elizabeth Fearon, James R. Hargreaves

**Affiliations:** London School of Hygiene & Tropical Medicine, London, UK

## Abstract

**Background:**

An epidemic of health disorders can be triggered by a collective manifestation of inappropriate behaviors, usually systematically fueled by non-medical factors at the individual and/or societal levels. This study aimed to (1) landscape and assess the evidence on interventions that reduce inappropriate demand of medical resources (medicines or procedures) by triggering behavioral change among healthcare consumers, (2) map out intervention components that have been tried and tested, and (3) identify the “active ingredients” of behavior change interventions that were proven to be effective in containing epidemics of inappropriate use of medical resources.

**Methods:**

For this systematic review, we searched MEDLINE, EMBASE, the Cochrane Library, and PsychINFO from the databases’ inceptions to May 2019, without language restrictions, for behavioral intervention studies. Interventions had to be empirically evaluated with a control group that demonstrated whether the effects of the campaign extended beyond trends occurring in the absence of the intervention. Outcomes of interest were reductions in inappropriate or non-essential use of medicines and/or medical procedures for clinical conditions that do not require them. Two reviewers independently screened titles, abstracts, and full text for inclusion and extracted data on study characteristics (e.g., study design), intervention development, implementation strategies, and effect size. Data extraction sheets were based on the checklist from the Cochrane Handbook for Systematic Reviews.

**Results:**

Forty-three studies were included. The behavior change technique taxonomy v1 (BCTTv1), which contains 93 behavioral change techniques (BCTs), was used to characterize components of the interventions reported in the included studies. Of the 93 BCTs, 15 (16%) were identified within the descriptions of the selected studies targeting healthcare consumers. Interventions consisting of education messages, recommended behavior alternatives, and a supporting environment that incentivizes or encourages the adoption of a new behavior were more likely to be successful.

**Conclusions:**

There is a continued tendency in research reporting that mainly stresses the effectiveness of interventions rather than the process of identifying and developing key components and the parameters within which they operate. Reporting “negative results” is likely as critical as reporting “active ingredients” and positive findings for implementation science. This review calls for a standardized approach to report intervention studies.

**Trial registration:**

PROSPERO registration number CRD42019139537

Contributions to the literature

- This review identifies the types, components, and combinations of interventions more likely to successfully initiate and sustain public behavior change in the context of complexity.
- It can inform practitioners’ decisions about designing, implementing, and reporting interventions to reduce inappropriate use/demand of medical interventions while researchers and funders can use this review to determine where research is needed.
- No community-based interventions were found in LMICs; interventions were limited to primary care settings or policy restrictions on the supply side (e.g., ban on over-the-counter purchases).
- There is a need for standardized reporting of intervention development, adaptation, and implementation to maximize generalisability and replicability.

## Background

Epidemics, which traditionally refer to a widespread occurrence of an infectious disease in a community at a particular time, have in recent years been used to describe large-scale public health issues caused by a shared pattern of human behaviors that impact public health and well-being. An epidemic of health disorders can not only be triggered by organisms that cause communicable diseases, such as bacteria, viruses, fungi, or parasites, but also by a collective manifestation of inappropriate behaviors, usually systematically fueled by non-clinical factors at the individual and/or societal levels. When medicines or medical procedures are used for conditions for which they should not be used, they are deemed as inappropriate use of medical interventions. For example, the World Health Organization and governments have warned about the recent spike in the use of prescription drugs [1] and cesarean sections [2] globally, which has formed an epidemic that has caused avoidable damage to individual health and introduced excessive burdens on health systems [3, 4].

There have been experiments with programs specifically designed to address factors driving the epidemics of inappropriate use of medical interventions*.* These countermeasures are often non-clinical behavioral change interventions targeting physicians and pharmacists as a point-of-entry for interventions and are designed to improve clinical practices and policies that restrict unnecessary dispensing [5, 6]. These programs usually employed educational materials (e.g., guidelines, lectures, workshops) [7, 8], auditing and feedback on prescribing practices [9–12], or computer-aided clinical decision support systems [13]. A 2005 Cochrane review concluded that, for interventions occurring on multiple levels to be effective, local barriers to change—including the role patients play in driving inappropriate demand—must be addressed [14]. Current interventions to address the pressure of inappropriate demands outside the clinical setting range from national mass media campaigns to local interventions targeted at smaller communities [15], aiming to influence the knowledge, attitudes, and practices towards medical use of the general public who have yet to become healthcare consumers: namely patients and caretakers of patients [15–17]. However, recent reviews highlighted that critical knowledge gaps exist in the evidence for engaging healthcare consumers as active decision-makers for appropriate medical use (as opposed to passive receivers of education materials) [18, 19]. Furthermore, the lack of evidence in the development of and evaluation of the impact of these interventions, especially in low- and middle-income countries (LMICs), complicates replication efforts [16, 17, 20].

The Behavioral Change Wheel (BCW) [21] and the behavior change techniques taxonomy volume 1 (BCTTv1) [22], developed by Michie and colleagues, facilitate researchers in organizing the content and components of behavioral interventions into nine intervention functions: *education, persuasion, incentivization, coercion, training, enablement, modeling, environmental restructuring, and restrictions* and assists them in translating specific techniques that were employed in a given intervention into change behaviors. Scientists have supported the use of BCW and BCTTv1 as a reliable and validated methodology that offers a common language for describing intervention components that can be used for the standardization of intervention content analysis and the development of interventions [23–25].

In this study, we aimed to (1) landscape and critically assess the evidence on non-clinical programs that reduce inappropriate or unnecessary use of medical interventions (i.e., medicines or medical procedures) by triggering behavioral change among healthcare consumers, (2) map out intervention components that have been tried and tested, and (3) identify the “active ingredients” of behavior change intervention programs that were proven to be effective in containing “epidemics of inappropriate use of medical interventions.”

## Methods

### Searches

For this systematic review, we searched MEDLINE, EMBASE, the Cochrane Library, and PsychINFO from the databases’ inceptions to May 2019, without language restrictions, for behavioral intervention studies. A search strategy was first developed for MEDLINE and adapted to other databases. The full-search strategy is detailed in Additional file 1. We searched for behavioral change interventions that aimed to reduce inappropriate or non-essential use of medical services or medicines that were driven by non-clinical factors and targeted health care consumers in the community, including primary care settings. For the purpose of this study, health care consumers included the public, patients, and caregivers (e.g., parents or guardians).

### Study inclusion and exclusion criteria

Inclusion and exclusion criteria used for all stages of the screening process are stated in Additional file 2. Studies had to be empirically tested by either randomized controlled trial (RCT), cluster-RCT (CRT), nonrandomized controlled trial (NCT), or interrupted times series (ITS) where the intervention time was clearly defined, and there were at least three data points both before and after the intervention, or quasi-experiments with a control group. To enable assessment of effectiveness in included interventions, this review excludes before/after evaluations of public campaigns or interventions that failed to employ a control group and therefore cannot show whether the effects of the campaign extended beyond trends occurring in the absence of the intervention. Outcomes of interest were reductions in inappropriate or non-essential use of medicines and/or medical procedures for clinical conditions that do not require them. Four major types of behaviors were identified, namely inappropriate antibiotic consumption (e.g., for viral infections or self-limiting conditions), elective cesarean section, demand for brand-name drugs that are available as generics, and non-medical use of prescription drugs, defined as “use without a prescription or use for reasons other than what the medication is intended for” [16, 26, 27]. Studies that focused only on change of knowledge or attitudes and did not report actual behavioral data were excluded. Studies mainly targeting clinicians, other healthcare staff, hospitals, inpatients, emergency care, or patients with mental health conditions were excluded. To create a distinction between interventions directed at health care consumers rather than providers, studies that aimed to modify clinical practices (e.g., prescribing) were excluded. Also, to differentiate behavior change interventions from therapies/treatments addressing mental health conditions such as addiction or depression, we excluded interventions for substance abuse, where inappropriate use was an outcome of a clinical condition, not a cause.

### Data extraction strategy

All titles retrieved from the searches were imported into Endnote referencing software. Duplicates were removed. Titles and abstracts were independently screened for inclusion by two reviewers (L.L and P.A.) and removed if deemed irrelevant. Both authors independently screened the full text (*n* = 347) of the remaining studies to assess eligibility. Substantial agreement was found at all three stages (> 90%). Disagreements were resolved through discussion among reviewers to achieve consensus; any further discrepancies about study inclusion were resolved through discussion with a third reviewer (E.F. or J.H). We also manually searched the bibliographies of all the included studies and reference lists of relevant systematic reviews to identify additional citations.

We extracted the data on study characteristics: the country where the study was conducted, type of inappropriate use, target population, study design (e.g., RCT, controlled pre- and post-study [CPP]), data collection methods (e.g., survey, interview, medical records), and, when focused on a population study, sampling methodology (e.g., cluster, convenience), primary or main outcome measure, and conclusions reported. We further examined reporting on intervention development/adaption, design, and implementation strategies. Additionally, we extracted underlying theoretical domains, effect size, and risk of bias by two independent review authors, who determined the domains within the Behavioral Change Wheel (BCW) and identified the “active ingredients” of the interventions according to BCTTv1. Data extraction sheets were based on the checklist from the Cochrane Handbook for Systematic Reviews [28]. The forms were modified after piloting on a sample of studies. When coding, we adopted the coding assumptions reported by Presseau et al. [25] that BCTs worked through targeting the behavior of health care consumers, or both the behavior of health care consumers and providers. We also assumed policy interventions and national campaigns were driven by governments and therefore coded governments as implementers for respective interventions. After the data extraction phase, we identified critical evidence gaps in evaluation data and processes of intervention development and implementation. We therefore conducted another round of targeted, investigative searches, involving citation and publication searches on first, last, and corresponding authors of selected interventions, seeking formative, process, and impact evaluation data.

### Study quality assessment

We conducted and reported the review in line with the Preferred Reporting Items for Systematic Reviews and Meta-Analyses statement (PRISMA). Risk of bias was assessed by two reviewers using the Effective Public Health Practice Project’s (EPHPP) Quality Assessment Tool for Quantitative Studies [29], which includes eight components (21 items): selection bias, study design, confounders, blinding, data collection methods, withdrawals or dropouts, intervention, and integrity. A rating of weak, moderate, or strong was given to each of the first six components, and these scores contributed to a global rating for the study. Qualitative data was assessed by the Critical Appraisal Skills Programme (CASP) checklist.

### Data synthesis on active ingredients

Using BCW domains and BCT taxonomies, we analyzed descriptions of all interventions and identified the commonly targeted aspects by looking at the frequency with which BCW domain and BCT of the interventions were incorporated in the studies. We also explored the nature and pattern of the use of these active ingredients across the different studies, and the associated magnitude of effect size. We descriptively reported the active ingredients and primary outcomes’ effect sizes at the study level, counting the number of times a BCW domain and a BCT had been identified across studies and in different types of use behaviors and presented a description of features of included interventions.

## Results

### Review statistics

**Fig. 1:**
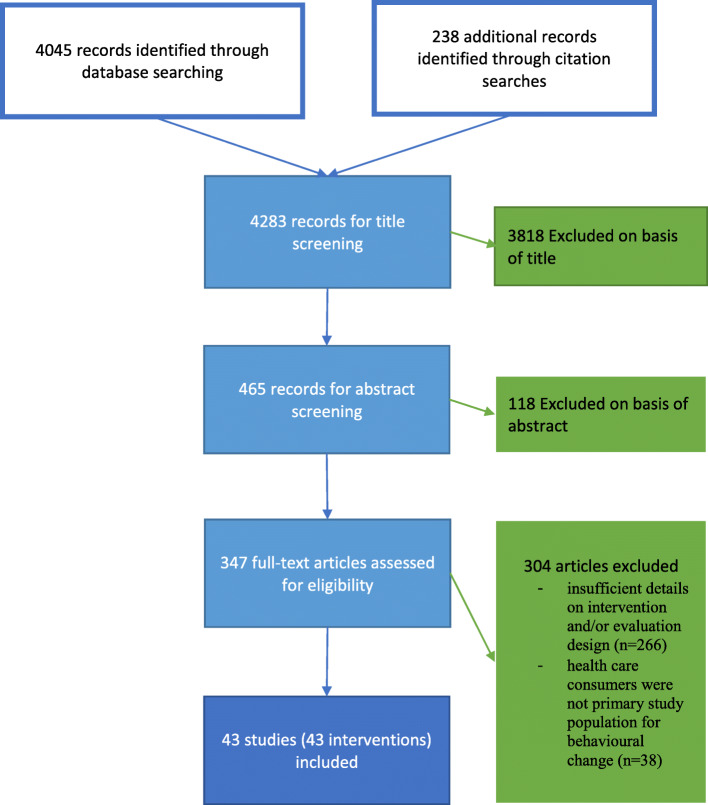
Flow diagram of systematic review search

Our systematic search of the literature yielded 4045 results through database searching and an additional 238 were identified through bibliography searches. After de-duplication and title and abstract screening, 347 references were assessed in full text. A flow diagram of the study selection process is shown in Fig. 1. Forty-three studies (representing 43 interventions, see Additional file 3)—conducted between 1994 and May 2019 and meeting inclusion criteria—were included in the systematic review. Twenty-five studied interventions focused on the reduction of antibiotic use—eight on elective cesarean section, four on the conversion from brand name drugs to generic equivalents, and six on nonmedical use of prescription drugs. Table 1 provides an overview of the included intervention studies for full-text extraction including intervention aims and components.

**Table 1:** An overview of the included studies: intervention aims, components, and reporting

	Context				Intervention elements										Implementation									
First author, year	Target illness/condition	Country	Last month of data collected	Setting	Target drivers/factors	Name	Slogan	Target audience	Healthcare providers	Healthcare consumers	BCT-provider	BCT-consumer	Behavioral Change Wheel	Theory-based	Intervention adaption/development	Implementation strategy	Implementer(s)	Unit of intervention	Dose/intensity	Design	Costs	Duration	Data sources	Formative or process evaluation studies
Inappropriate use of antibiotics																								
Belongia, 2001	RTIs	USA	June 1998	Community and primary care setting	Knowledge (including awareness), cultural, and doctor-patient relationship	–	None	Community and healthcare providers	Physician education (parent education pamphlets, parent information sheets, a sample letter, “prescription pad,” CDC fact sheets	Public education materials: programs, pamphlets and posters, presentations and “Cold kits”	4.1 4.2 5.1 8.2 12.5	4.1 4.2 5.1 8.2 12.5	Education	–	Yes	Yes	Yes	Community	Partially reported	NR	NR	4 months	Medical records + self-reports, lab testing	
Belongia, 2005	Not specified	USA	December 2003	Community	Knowledge (including awareness)	Wisconsin antibiotic resistance network	“There’s no excuse for overuse!” and “Get smart about antibiotics!”	Community and healthcare providers	Physician education (mailings, susceptibility reports, practice guidelines, satellite conferences, and presentations)	Mass media campaign (television, radio, newspapers, press conference; paid ad); Patient education materials	4.1 4.2 5.1 12.5	4.1 4.2 5.1 12.5	Education	**–**	Yes	Yes	Yes	Community	Yes	Access expired	NR	5 years	Medical records	–
Bernier, 2014	Not specified	France	December 2010	Community	Knowledge (including awareness)	–	“Antibiotics are not automatic!” and “antibiotics, used unnecessarily, lose their potency!”	Community	Guidelines, seminars, academic detailing, letters	Pamphlets and posters, print media, radio, television, website	4.1 4.2 5.1 12.5	4.1 4.2 5.1 12.5	Education	–	NR	NR	Yes	Community	NR	NR	NR	6 months (ongoing)	Medical records	–
Cebotarenco, 2008	RTIs	Moldova	March 2004	School setting	Knowledge (including awareness) peer	–	None	Community-students and guardians	–	Peer-education, parents’ meetings, booklet, vignette video, newsletter, poster, and poster contest	–	4.1 4.2 6.1 12.2	Education	Social cognitive theory	Yes	Yes	Yes	Community	Yes	Yes	NR	1 year	Self-reports	–
Finkelstein, 2001	RTIs	USA	December, 1998	Community & primary care setting	Knowledge (including awareness), doctor-patient relationship, peer leader	–	–	Community and healthcare providers	Guideline dissemination, small-group education, educational materials, and prescribing feedback.	Educational materials for parents by mail and in primary care practices, pharmacies, and childcare settings	2.2 3.2 4.1 4.2 5.1 8.2 9.1	4.1 4.2 5.1 8.2 9.1	Education	–	Yes	Yes	Yes	Community	NR	NR	NR	1 year	Medical records	[30]
Finkelstein, 2008	RTIs	USA	August 2003	Community	Knowledge (including awareness), doctor-patient relationship	Reducing antibiotics for children in Massachusetts (REACH Mass)	None	Community and healthcare providers	Guideline dissemination, small-group education, educational materials, “prescription pad”, and prescribing feedback.	Educational materials for parents by mail and in primary care practices, pharmacies, and childcare settings	2.2 3.2 4.1 4.2 5.1 8.2	4.1 4.2 5.1 8.2	Education	Social marketing	Yes	Yes	Yes	Community	Partially reported	NR	NR	3 winters (Oct-March)	Medical records	[30]
Formoso, 2013	RTIs	Italy	March 2012	Community	Knowledge (including awareness), cultural, and doctor-patient relationship	Antibiotics, solution or problem	“Antibiotics, solution or problem?”	Community and healthcare providers	a newsletter on local AMR. campaign materials (highlighting how to deal with patients’ expectations, occurrence of AMR and of side effects.)	mass media spaces (television, radio, newspapers) written materials (brochures, posters, newsletters)	4.1 4.2 5.1 5.2 12.5	4.1 4.2 5.1 5.2 12.5	Education/persuasion	Social marketing	Yes	Yes	Yes	Community	Partially reported	Access expired	$60,800	4 months	Medical records + self-reports	–
Fuertes, 2010	Not specified	Canada	December 2008	Community	Knowledge (including awareness)	Do bugs need drugs?	None	Community and healthcare providers	Television campaign	Television campaign	4.1 4.2 5.1 5.2 8.2	4.1 4.2 5.1 5.2 8.2	Education	–	NR	Yes	Yes	Community	NR	NR	NR	5 months	Medical records	–
Gonzales, 2004	RTIs	USA	February 2002	Community & primary care setting	Knowledge (including awareness) and doctor-patient relationship	Minimizing antibiotic tesistance in Colorado	Be SMART about antibiotics	Community and healthcare providers	Antibiotic prescribing profiles and practices guidelines	Waiting room materials, examination room posters; mailing campaign packets: household- and office-based patient education materials	1.3 12.5	4.1 4.2 5.1 9.1 12.5	Education	–	Yes	Yes	Yes	Community	Access expired	Access expired	NR	1 year	Medical records	[31]
Gonzales, 2005	RTIs	USA	February 2002	Community & primary care setting	Knowledge (including awareness) and doctor-patient relationship	Minimizing antibiotic resistance in Colorado	Be SMART about antibiotics	Community and healthcare providers	antibiotic prescribing profiles and practices guidelines	Waiting room materials, examination room posters; mailing campaign packets: household- and office-based patient education materials	1.3 12.5	4.1 4.2 5.1 9.1 12.5	Education	–	Yes	Yes	Yes	Community	Access expired	Access expired	$63,745	1 year	Medical records	(see Gonzales, 2004)
Gonzales, 2008	Not specified	USA	December 2003	Community	Knowledge (including awareness)	Minimizing antibiotic resistance in Colorado	“Get amart: use antibiotics wisely.” and *“Use antibio´ ticos solo si un doctor se lo receta”*	Community and healthcare providers	Primary care physicians	Mass media campaign, educational events and written educational materials	4.1 4.2 5.1 12.5	4.1 4.2 5.1 12.5	Education	Social marketing	Yes	Yes	Yes	Community	Yes	Yes	$196,710	4 months	Medical records + self-reports	–
Hennessy, 2002	RTIs	USA	December 2000	Community	Knowledge (including awareness)	–	–	Community and healthcare providers	Workshops and follow-up visits	Printed information and newsletters	4.1 4.2	4.1 4.2 5.1	Education	–	Yes	Yes	Yes	Community	Access expired	Access expired	NR	6 months	Medical records + lab testing + self-reports	–
Kliemann, 2016	Not specified	Brazil	December 2012	Community	Socioeconomic determinants; access to non-prescription antibiotics	–	–	Community and healthcare providers	Restriction on sale of antibiotics without prescription	Restriction on sale of antibiotics without prescription	12.1	12.1	Restriction, environmental restructuring	–	NA	Yes	Yes	Community	NA	NA	NA	Ongoing	Medical records	–
Lambert, 2007	RTIs	UK	February 2005	Community	Knowledge (including awareness)	–	Antibiotics – tracking down the trust	Community and healthcare providers	Professional education and prescribing support	Mass media with printed materials	4.1 8.2 12.5	4.1 8.2 12.5	Education	–	NR	Yes	Yes	Community	NA	Partially reported	£25,000	2 winters	Medical records + self-reports	–
Lee, 2017	RTIs	Singapore	Not specified	Primary care setting	Knowledge (correcting misconceptions)	–	–	Community - patients	–	Educational pamphlets and verbal counseling	–	4.1 4.2	Education	–	NR	NR	Yes	Individual	NR	NR	NR	2 weeks	Medical records	–
Mainous, 2009	Not specified	USA	June 2008	Community	Knowledge (including misconceptions); cultural	“Solo Con Receta” (only with a prescription)	–	Community	–	Culturally sensitive community intervention with multiple media sources	–	4.1 5.1	Education	–	NR	Yes	Yes	Community	Partially reported	NR	NR	9 months	Medical records + self-reports	–
McNulty, 2010	RTIs	UK	January 2009	Community & primary care setting	Knowledge (correcting misconceptions)	–	–	Community - patients	NICE guidance on the primary care management of common, acute, self-limiting RTIs	Three posters displayed in magazines and newspapers	4.1 4.2 8.2	4.1 4.2	Education	–	NR	NR	Yes	Individual	NR	Yes	NR	2 months	Self-reports	[32]
Perz, 2002	RTIs	USA	April 1999	Community	Knowledge (including awareness); peer	–	Antibiotics and your child	Community and healthcare providers	Educating peer leader presentations	Public education via printed material	4.1 4.2	4.1 4.2 8.2	Education	–	Yes	Yes	Yes	Community	Partially reported	Partially reported	NR	1 year	Medical records	–
Sabuncu, 2009	RTIs	France	December 2007	Community	Knowledge (including awareness)	Keep antibioticsworking	“Les antibiotiques c’est pas automatique” (“Antibiotics are not automatic”)	Community	Guidelines, seminars, academic detailing, letters	Pamphlets and posters, print media, radio, television, website	4.1 4.2 5.1 12.5	4.1 4.2 5.1 12.5	Education	–	NR	NR	Yes	Community	NR	NR	NR	5 years	Medical records	(see Bernier, 2014)
Santa-Ana-Tellez, 2013	Not specified	Brazil and Mexico	June 2012	Community	Access to non-prescription antibiotics	–	–	Community and healthcare providers	Restriction on sale of antibiotics without prescription in pharmacies, and introduction of fine on owners of pharmacies for non-compliance.	Restriction on sale of antibiotics without prescription	12.1 14.2 (only Mexico)	12.1	Restriction, coercion, environmental restructuring	–	NA	Yes	Yes	Community	NA	NA	NA	Ongoing	Medical records	[33–36]
Santa-Ana-Tellez, 2015	Not specified	Brazil and Mexico	March 2012	Community	Access to non-prescription antibiotics	–	–	Community and healthcare providers	Restriction on sale of antibiotics without prescription in pharmacies, and introduction of fine on owners of pharmacies for non-compliance.	Restriction on sale of antibiotics without prescription	12.1 14.2 (only Mexico)	12.1	Restriction, coercion, environmental restructuring	–	NA	Yes	Yes	Community	NA	NA	NA	Ongoing	Medical records	(see Santa-Ana-Tellez, 2013)
Taylor, 2005	RTIs	USA	April 2002	Primary care setting	Knowledge, doctor-patient relationship	–	Puget Sound Pediatric Research Network	Community - parents and children	-	Educational pamphlets and a video	–	4.1 9.1	Education	–	Yes	Yes	Yes	Community	NR	NR	NR	1 year	Medical records	–
Trepka, 2001	RTIs	USA	August 1998	Community & primary care setting	Knowledge (including awareness), cultural, and doctor-patient relationship	–	Your child and antibiotics	Community and healthcare providers	“Grand rounds” presentations, small-group academic detailing, and distribution of written materials (clinical practice guidelines, clinical fact sheets, and samples of patient education materials.)	Public education materials: programs, pamphlets, and posters, presentations and newspapers	4.1 4.2 5.1 8.2 12.5	4.1 4.2 5.1 8.2 12.5	Education	–	Yes	Yes	Yes	Community	Partially reported	NR	NR	4 months	Self-reports	–
Wirtz, 2013	Not specified	Chile, Colombia, Venezuela, Mexico	September 2009	Community	Access to non-prescription antibiotics	–	–	Community and healthcare providers	Restriction on sale of antibiotics without prescription	Restriction on sale of antibiotics without prescription	12.1	12.1	Restriction, coercion, environmental restructuring	–	NA	Yes	Yes	Community	NA	NA	NA	Ongoing	Medical records	[33–36]
Wutzke, 2007	RTIs	Australia	August 2004	Community & primary care setting	Knowledge, doctor-patient relationship; peer	The NPS common colds community campaign	“Common colds need common sense: they don’t need antibiotics.”	Community and healthcare providers	Prescription pads, patient information leaflets, prescribing software. newsletters, prescribing feedback, educational visiting, clinical audit with feedback and case studies (paper and peer group discussion).	Mass media activity using billboards, television, radio, and magazines and small grants to promote local community education	2.2 3.1 4.1 4.2 8.2 12.5	4.1 4.2 8.2 12.5	Education/persuasion	–	Yes	Yes	Yes	Community	Partially reported	Yes	NR	6 years	Medical records + self-reports	–
Demand of brand name drugs																								
Beshears, 2013	Not specified	USA	October 2014	Community	Knowledge (including awareness), peer influence	–	–	Community - union members	–	Informational letters with or without a testimonial from person with/without shared union affiliation	–	8.2 9.1 10.1 10.2	Education, persuasion	–	NR	Yes	Yes	Individual	Partially reported	NR	NR	1 letter	Medical records	–
O'Malley, 2006	Not specified	USA	December 2003	Community	Knowledge (including awareness), incentives	–	–	Community and healthcare providers	Free generic drug samples, physician financial incentives	Member mailings, advertising campaigns	3.2 4.1 8.2 10.1 10.2 12.5	4.1 8.2 10.1 10.2 12.5	Education, incentivization	–	NR	Yes	Yes	Community	NR	NR	NR	4 years	Medical records	–
Sedjo, 2009	Not specified	USA	December 2007	Community	Knowledge (including awareness), incentives	–	–	Community – health plan enrollees	–	Targeted messaging to raise awareness regarding lower-cost generic alternatives (a phone call and quarterly letters)	–	4.1 8.2 10.1 10.2	Education, incentivization	–	NR	Yes	Yes	Individual	NR	NR	NR	1 call and quarterly mails	Medical records	–
Vallès, 2003	Not specified	Spain	February 2000	Primary care setting	Knowledge (including awareness)	–	–	chronic disorders patients who attended general practices	–	Verbal information and handout materials on advantages and disadvantages of generic equivalents and brand-name drugs	–	4.1 8.2 9.2	Education	–	NR	Yes	Yes	Individual	NR	NR	NR	1 session	Medical records	–
Non-medical use of prescription drugs																								
Hasak 2018	Pain management (short-term	USA	September, 2017	Community	Knowledge (including awareness), enabling	–	–	–	–	Information brochure, website		4.1 4.2 5.1 5.2 12.1	Education; enablement	–	Yes	Yes	Yes	Individual	Yes	Yes	NR	2 times	Self-reports	[37]
Lawrence, 2019	Pain management (short-term	USA	January 2019	Community	Knowledge (including awareness), enabling	–	–	–	–	Information brochure, video, Deterra bags		4.1 4.2 5.1 5.2 12.1 12.5	Education; enablement; environmental restructuring;	–	Yes	Yes	Yes	Individual	Yes	Yes	Partially reported ($5–7 per bag)	1 time	Medical records, self-reports	[38]
Maughan, 2016	Pain management (short-term	USA	October 2015	Community	Knowledge (including awareness), enabling	–	–	–	–	Information brochure, study hotline		4.1 4.2 5.1 5.2 12.1 12.5	Education; enablement; environmental restructuring;	–	NR	Yes	Yes	Individual	Yes	NR	NR	1 time	Self-reports	
Rose, 2016	Pain management (short-term	Canada	April 2015	Community	Knowledge (including awareness), enabling	–	–	–	–	Information brochure		4.1 4.2 5.1 5.2 12.1	Education; enablement	–	Yes	Yes	Yes	Individual	Yes	Yes	NR	1 time	Self-reports	
Spoth, 2008	Not specified	USA	December 2002	School setting	Enhance protective factors Family dynamics	Strengthening Families Program (ISFP) and Life Skills Training (LST)	–	Community - students	–	Universal preventive interventions implemented during middle school (strengthening families program and life skills training)	–	3.1 12.2	Education; enablement; environmental restructuring;	Social development model	NR	Yes	Yes	Individual	NR	NR	NR	6 2-h sessions + 1 family follow-up + boosters (cohort)	Self-reports	[39–44]
Spoth, 2013	Not specified	USA	December 2011	School setting	Enhance protective factors Family dynamics	Strengthening Families Program (ISFP) and Life Skills Training (LST)	–	Community - students	–	Universal preventive interventions implemented during middle school (strengthening families program and life skills training)	–	3.1 12.2	Education; enablement; environmental restructuring;	Social development model	NR	Yes	Yes	Individual	NR	NR	NR	6 2-h sessions + 1 family follow-up + boosters (cohort study 1:1993–2008; study 2: 1998–2011)	Self-reports	(see Spoth, 20080)
Elective cesarean section																								
Eden, 2014	Experienced previous cesarean birth	USA	May 2007	Community & primary care settings	Knowledge (including awareness), enabling	–	–	Community - pregnant women with one previous cesarean birth	–	Evidence-base information brochure or facilitated decision analysis	–	4.1 5.1 9.2	Education; enablement	–	Yes	Yes	Yes	Individual	NR	NR	NR	1 session	Medical records + self-reports	–
Fraser, 1997	Experienced previous cesarean birth	Canada	November 1994	Primary care setting	Knowledge (including awareness), Predisposing, enabling and reinforcing factors	–	–	Community - pregnant women with one previous cesarean birth	–	Educational pamphlet, prenatal education, and peer support program	–	3.3 4.1 5.1	Education; enablement	The PRECEDE-PROCEED model	NR	Yes	Yes	Individual	NR	NR	NR	2 sessions	Medical records + self-reports	–
Hassani, 2016	Not specified	Iran	**NR**	Primary care setting	Knowledge (including awareness	–	–	Community - primiparous pregnant women	–	Instructional sessions in the form of speech, group discussions, questions and answers, and presentations		4.1	Education	Health belief model	NR	Yes	Yes	Individual	NR	NR	NR	6 sessions–50–60 min/session	Self-reports	–
Montgomery, 2007	Experienced previous cesarean birth	UK	August 2006	Primary care setting	Knowledge (including awareness), enabling	–	–	Community - pregnant women with one previous cesarean birth	–	Information program and facilitated decision analysis	–	4.1 5.1 9.2 9.2	Education; enablement	–	Yes	Yes	Yes	Individual	NR	NR	NR	10 weeks	Medical records + self-reports	[45–49]
Navaee, 2015	Fear of childbirth	Iran	NR	Primary care setting	Knowledge (including awareness), emotions	–	–	Community - primiparous pregnant women	–	Education through role play about advantages and disadvantages	–	4.1 4.2 6.1 9.2	Education; modeling	–	NR	Yes	Yes	Individual	NR	NR	NR	1 session–90 min	Self-reports	–
Sharifirad, 2013	Primiparous pregnant women	Iran	NR	Primary care setting	Knowledge (including awareness), family dynamics	–	–	Community—spouses of primiparous pregnant women	–	Educational session about mechanism of natural vaginal and cesarean deliveries as well as their advantages and disadvantages.	–	3.1 4.1 5.1 9.2	Education; enablement	–	NR	Yes	Yes	Individual	NR	NR	NR	1 session –90 min	Self-reports	–
Shorten, 2005	Experienced previous cesarean birth	Australia	May 2003	Primary care setting	Knowledge (including awareness), enabling	–	–	Community—pregnant women with one previous cesarean birth	–	Information materials and facilitated decision analysis	–	4.1 5.1 9.2	Education; enablement	–	Yes	Yes	Yes	Individual	NR	NR	NR	1 session	Medical records + self-reports	[50]
Valiani, 2014	Primiparous pregnant women	Iran	NR	Primary care setting	Knowledge (including awareness)	–	–	Community—primiparous pregnant women	–	Childbirth workshops	–	4.1 4.2 5.1 6.1 9.2	Education; enablement	–	NR	Yes	Yes	Individual	NR	NR	NR	3–4 h/week	Medical records	–

Note: *NR* not reported, *RTIs* respiratory tract infections, *GP* general practitioner, *CS* elective aesarean section

### Study characteristics

All included studies were published in English. Twenty-four in North America (excluding Mexico; USA: *n* = 21, Canada: *n* = 3), four in Latin America (Chile, Colombia, Venezuela, Brazil, and Mexico), four in the Middle East (Iran), eight in Europe (France, UK, Italy, Spain, and Moldova), three in East Asia and Pacific (Australia and Singapore), and none from sub-Saharan Africa, South Asia, or the Caribbean. The imbalance between high-income countries (HICs) and low- and middle-income countries (LMICs) is apparent when characterizing types of inappropriate use. Multifaceted interventions are scarce and limited to HICs while interventions in LMICs were limited to primary care settings or policy restrictions (on over-the-counter purchases) with zero community-based programs identified. No studies from LMICs focused on demands for brand-name drugs or non-medical use of prescription drugs.

### Study design

The included studies consisted of 18 RCTs and five NCTs, eight ITS, and 12 quasi-experimental studies. These studies varied in their quality, methodological design, and implementation. Twenty-four studies reported longitudinal data; the rest employed cross-sectional study designs. All were outcome evaluation studies. In terms of data collection methods for evaluation, 23 studies employed surveys and 30 utilized medical record data—these were not mutually exclusive. Four studies reported cost data. One study employed interviews as part of the intervention procedure, but not for evaluation purposes [51]. No qualitative data were reported in the initial included studies; we therefore conducted a targeted, investigative search on the selected interventions, but only located minimal formative data on some of the studies [30, 45–47, 50]. One UK-based project that aimed to improve the decision-making around mode of delivery among pregnant women published comprehensive implementation research data from pilot results [48] and study protocol [47] to outcome and economic evaluation [45, 46, 49, 52, 53]. Table 2 presents a summary of the key characteristics of each study measuring behavioral outcomes and reported formative and relevant evaluation data of the included interventions.

**Table 2:** Summary of findings of included studies measuring changes behavioral outcomes

First author, year	Study design		Study population	Study sample size	Primary outcome(s)	Change in intervention group	Change in control group	Effect size (95% CI)	*P* value	Effective in changing public behaviors	Quality appraisal
Belongia, 2001	NCT	Longitudinal	Physicians and public	111 facilities, 664 children	Pediatric antibiotic prescribing in child care facilities	Baseline: 57.6%; post-intervention: 59.5% of initial visits	Baseline: 60.1%; post-intervention 61.5% of initial visits	NR	Baseline: *P* = 0.56; post-intervention: *P* = 0.66	No	Weak
Belongia, 2005	CPP	Longitudinal	Parents and primary care clinicians	4115 primary care physicians	Change in annual antimicrobial prescribing rate	− 20.4%	− 19.8%	− 0.6%	NR	No	Moderate
Bernier, 2014	ITS	Longitudinal	French citizens covered by NHI	Not reported	Change in antimicrobial prescribing rate	NA	NA	− 30% (− 36.3 to − 23.8%)	*P* < 0.001	Mixed	Strong
Cebotarenco, 2008	CPP	Cross-sectional	Students and parents	~6302 people	No antibiotic use for cold and flu	Students: a 33.7% net increase in no antibiotic use; Adults: a 38.0% net increase in no use	Students − 0.4%; adults +0.1%	Students 3.694 (CI 2.516 to 5.423); adults 5.541 (CI 4.559 to 6.733)	*P* < 0.0001	Yes	Weak
Finkelstein, 2001	RCT	Longitudinal	Physicians and parents	8815 children	Antibiotics dispensed per person-year of observation among children	3 to < 36 months (− 18.6%), 36 to < 72 (− 15.0%)	3 to < 36 months (− 11.5%), 36 to < 72 (− 9.8%)	3 to < 36 monnths (− 16%), 36 to < 72 (− 12%)	3 to < 36 months (*P* < 0.001), 36 to < 72 (*P* < 0.001)	Yes	Strong
Finkelstein, 2008	RCT	Longitudinal	Physicians and parents	223,135 person/years	Antibiotics dispensed per person-year of observation among children	3 to < 24 months (− 20.7%), 24 to < 48 (− 10.3), 48 to < 72 (− 2.5)	3 to < 24 months (− 21.2), 24 to < 48 (− 14.5), 48 to < 72 (− 9.3)	3 to < 24 months (− 0.5), 24 to < 48 (− 4.2), 48 to < 72 (− 6.7)	3 to < 24 months (*P* = 0.69), 24 to < 48 (*P* < 0.01), 48 to < 72 (*P* < 0.0001)	Mixed	Strong
Formoso, 2013	NCT	Longitudinal	Modena and Parma, Emilia-Romagna region	1,150,000 residents	Antibiotic prescription rate	− 11.9	− 7.4	− 4.3% (− 7.1 to − 1.5%)	*P* = 0.008	Yes	Strong
Fuertes, 2010	ITS	Longitudinal	Population in British Columbia, Canada	Not reported	Antibiotic utilization rate	− 5.8%	NA	NR	NR	No	Strong
Gonzales, 2004	NCT	Longitudinal	Medicare enrollees with acute respiratory tract infections (ARIs)	4270 patient visits	Decreased antibiotic prescription rates	− 5%	− 2%	NR	*P* = 0.79	No	Moderate
Gonzales, 2005	NCT	Longitudinal	Children with pharyngitis and adults with acute bronchitis	Baseline: 10128 patients Study: 9586 patients	Decreased antibiotic prescription rates	Children: − 4% Adults: − 24%	Children: − 2% at local control; 1% at distant control; Adults: − 10% at local control; − 6% at distant control	NR	Children: *P* = 0.18, *P* = 0.48 compared with distant and local control; Adults: *P* < 0.002 and *P* = 0.006, for distant and local control	Mixed	Moderate
Gonzales, 2008	NCT	Longitudinal	Mothers of young children and primary care physicians	922 households, 1.38+ million antibiotic prescriptions	Net change in antibiotic dispensed per 1000 persons	–	–	− 3.8% in retail pharmacy antibiotic dispenses and − 8.8% in managed care organization (MCO)-associated dispenses	*P* = 0.30 for public, *P* = 0.03 for MOC members	Mixed	Strong
Hennessy, 2002	NCT	Longitudinal	Medical providers and community	10,809	Antibiotic utilization	− 31% (*P* ≤ 0.01)	− 10% (*P* ≥ 0.05)	− 21%	NR	Mixed	Moderate
Kliemann, 2016	ITS	Longitudinal	Residents of Sao Paulo	41,262,199	Antibiotic utilization	− 1.616 DID	NA	NR	*P* = 0.002	Yes	Moderate
Lambert, 2007	CPP	Longitudinal	Communities in North East of England	Not reported	Per person, per clinic visit	Initial: − 31% Expanded: − 35%	NA	NR	*P* < 0.01	Mixed	Weak
Lee, 2017	RCT	Cross-sectional	Adult patients	914 patients	Antibiotic prescriptions	20.6%	17.7%	1.20 (0.83–1.73)	*P* = 0.313	No	Weak
Mainous, 2009	QE (controlled post-test)	Cross-sectional	Latino adults	500 adults	Use of non-prescription antibiotics	1.3%	3.2%	NR	*P* = 0.90	No	Weak
McNulty, 2010	CPP	Cross-sectional	Adult ≥ 15	Pre= (1999); post (1830)	Antibiotic use without professional advice	− 0.5%	0%	NR	NR	No	Weak
Perz, 2002	CPP	Longitudinal	Children < 15	464200 person-years	Antibiotic prescription rates	Year 3:19%	Year 1: 8%	11% (8–14%)	*P* < 0.001	Yes	Moderate
Sabuncu, 2009	ITS	Longitudinal	French citizens covered by NHI	Not reported	Change in winter antibiotic prescribing rate (Oct to Mar)	NA	NA	− 26.5% (− 33.5 to − 19.6%)	< 0.0001	Yes	Strong
Santa-Ana-Tellez, 2013	ITS	Longitudinal	Populations in Mexico and Brazil	Not reported	OTC antibiotics consumption	Brazil = − 1.35; Mexico = − 1.17	NA	NR	Brazil *P* < 0.01; Mexico P < 0.001	Mixed	Strong
Santa-Ana-Tellez, 2015	ITS	Longitudinal	Populations in Mexico and Brazil	Not reported	Seasonal variation in total Penicillin use	Brazil = 0.077; Mexico = − 0.359	NA	Brazil = 0.077 (-1.142 to 1.297); Mexico = -0.359 (-0.613 to -0.105)	Brazil *P* > 0.05; Mexico *P* < 0.01	Mixed	Strong
Taylor, 2005	RCT	Cross-sectional	Parent/child dyads	499 children	Total no. of prescriptions for antibiotics	2.2 ± 2.6	2.5 ± 2.9	NR	*P* = 0.23	No	Weak
Trepka, 2001	CPP	Cross-sectional	Physicians and public	365 children	Expected an antibiotic for their child and did not receive one and brought their child to another physician because they did not receive an antibiotic	Expected an antibiotic for their child and did not receive one: − 5.1% brought their child to another physician because they did not receive an antibiotic: − 2.9%	Expected an antibiotic for their child and did not receive one: 3.2% brought their child to another physician because they did not receive an antibiotic: 1.6%	Expected an antibiotic for their child and did not receive one: − 8.4% (− 13.9 to − 2.8); brought their child to another physician because they did not receive an antibiotic: − 4.5% (− 8.0 to – 0.9), they did not receive an antibiotic: 1.6%	Expected an antibiotic for their child and did not receive one: *P* = 0.003 brought their child to another physician because they did not receive an antibiotic: *P* = 0.02	Yes	Weak
Wirtz, 2013	ITS	Longitudinal	Chile, Colombia, Venezuela, Brazil	Not reported	OTC antibiotics consumption	Colombia: − 2.4DID; Chile: − 3.8 DID; Venezuela: + 5.39DID and Mexico: − 2.4DID	NA	Colombia: − 1.00; Chile: − 5.56; Venezuela: opposite impact; Mexico: no difference	Colombia: *P* = 0.001; Chile: *P* < 0.05	Mixed	Moderate
Wutzke, 2007	ITS	Longitudinal	Australian community	Not reported	Change in use of antibiotics	− 3.40%	NA	1.3–5.5	< 0.05	Yes	Moderate
Beshears, 2013	RCT	Cross-sectional	union members	5498 adults	Conversion rate to lower-cost alternatives	Unaffiliated testimonial group 11.3%; Affiliated testimonial group 11.7%	12.20%	NR	NR (insignificant)	No	Moderate
O'Malley, 2006	QE (matched controlled)	Longitudinal	Adult patients	9790064 claims	Generic dispensing rate	Mailing: − 4.94; Advertising: − 0.13; Generic sampling: − 0.02; physician incentive: − 0.33	Doubling co-payment for brand-name drugs: 8.60	NR	*P* > 0.05	No	Moderate
Sedjo, 2009	QE	Longitudinal	Consumer-directed health care enrolees	4026 people	Conversion rate to lower-cost alternatives	0.30%	9.30%	29.82 (4.41–201.93)	*P* < 0.05	Yes	Moderate
Vallès, 2003	RCT	Longitudinal	Patients taking medications for chronic disorders	4620 patients	Evolution of the percentage of generic prescribing	5.10% (1999–2000)	1.90% (1999–2000)	NR	*P* < 0.001	Yes	Strong
Hasak 2018	QE	Cross-sectional	Postoperative patients	258 patients	Self-reported proper opioid disposal	28 (22)	14 (11)	NR	*P* = 0.02	Yes	Weak
Lawrence, 2019	RCT	Cross-sectional	Parents of postoperative patients	202 caregivers	Self-reported proper opioid disposal	66 (71.7)	50 (56.2)	15.5 (1.7 to 29.3)	*P* = 0.03.	Yes	Moderate
Maughan, 2016	RCT	Cross-sectional	Postoperative patients	79 patients	Self-reported proper opioid disposal	52% (16/31)	30% (8/27)	NR	*P* = 0.11.	No	Weak
Rose, 2016	QE	Cross-sectional	Postoperative patients	87 patients	Self-reported proper opioid disposal	12 (27%)	2 (5%)	22% (5 to 38)	*P* = 0.005	Yes	Weak
Spoth, 2008	RCT	Longitudinal	Late adolescents and young adults	2651 (study 2 on prescription drugs)	Self-reported lifetime prescription drug misuse overall	11th graders: 3.9%; 12th graders: 7.7%	11th graders: 7.7%; 12th graders: 10.5%	NR	11th graders: *P* < 0.01; 12th graders: *P* < 0.1	Yes	Weak
Spoth, 2013	RCT	Longitudinal	Late adolescents and young adults	Study 1: 667 students; Study 2: 2127 students	Self-reported lifetime prescription drug misuse overall	Study 1- 5.4; Study 2- 2.5 in age 21, 4.4 in age 22, 6.3 in age 25.	Study 1- 15.5; Study 2- 6.5 in age 21, 8.9 in age 22, 9.4 in age25.	Study 1: 65%; Study 2: 62% in age 21, 51% in age 22, 33% in age 25.	Study 1-*P* < 0.01; Study 2- age 21, *P* = 0.015, age 22, *P* = 0.019, age 25, *P* = 0.064	Yes	Weak
Eden, 2014	RCT	Cross-sectional	Pregnant women with previous cesarean	131 women	MoD (vaginal)	41%	37%	NR	*P* = 0.724	No	Weak
Fraser, 1997	RCT	Cross-sectional	Pregnant women with previous cesarean section	1275 women	MoD (vaginal)	53%	49%	1.1 (1.0 to 1.2)	*P* > 0.05	No	Weak
Hassani, 2016	QE	Cross-sectional	Primiparous women	60 women	MoD (vaginal)	30%	10%	NR	NR	Yes	Weak
Montgomery, 2007	RCT	Cross-sectional	Pregnant women with previous cesarean section	742 women	MoD (vaginal)	Decision analysis group: 37%; Info: 29%	Usual care: 30%	Info v. usual care: 0.93 (0.61,1.41) Decision v. usual care: 1.42 (0.94,2.14)	*P* > 0.9 *P* = 0.22	No	Strong
Navaee, 2015	RCT	Cross-sectional	Primiparous women	67 women	MoD (vaginal)	62.9%	43.8%	NR	*P* = 0.117	No	Weak
Sharifirad, 2013	RCT	Cross-sectional	Pregnant women and partners	88 women and partners	MoD (vaginal)	71.5%	50.0%	NR	*P* < 0.05	Yes	Weak
Shorten, 2005	RCT	Cross-sectional	Pregnant women with previous cesarean section	227 women	MoD (vaginal)	VD: 49.2%	CS: 50.8%	NR	NR	No	Weak
Valiani, 2014	RCT	Cross-sectional	Pregnant women and partners	180 women and partners	MoD (vaginal)	Mothers alone intervention = 60%; Couples = 56.7%	26.7%	NR	*P* = 0.017	Yes	Weak

Notes: *CS* elective cesarean section, *CPP* controlled pre- and post-study, *NA* not applicable, *NR* not reported, *PDMO* prescription drug misuse overall, *NCT* nonrandomized controlled trial, *OTC* over-the-counter purchases, *MoD* mode of delivery, *RCT* randomized controlled trial, *VD* normal vaginal delivery

### Study quality assessment

Study quality varied by domain assessed based on the primary behavioral outcomes (Additional file 4). There were 11 studies of overall strong quality, 12 of overall moderate quality, and 20 of overall low quality. In order to provide an overview of the entire literature, no studies were excluded based on their methodological quality. The majority of behavior outcomes were derived from medical records, leaving minimal room for reporting errors with the exception that some only relied on self-reported data for evaluation.

### Active ingredients of the behavior change interventions

**Fig. 2:**
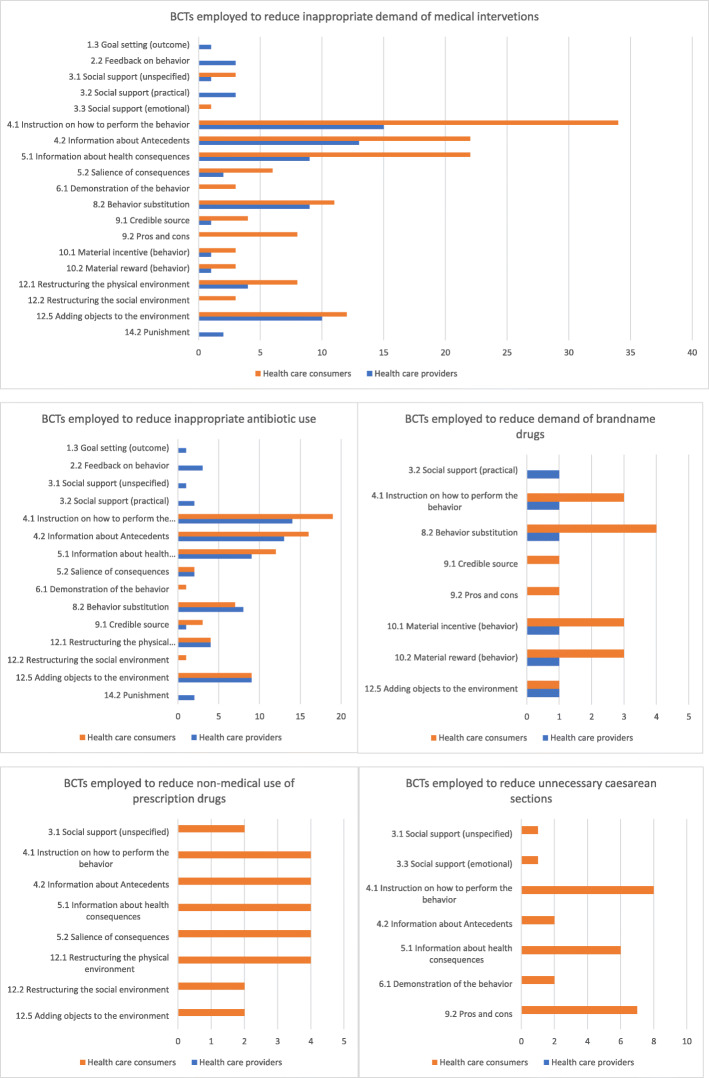
Frequency distribution of behavior change techniques (BCTs) coded for 43 interventions

All of the interventions utilized multiple behavior change techniques (BCTs) with a primary aim to improve health care consumers’ behavior. Table 3 presents the features of all the included interventions; the frequency distributions of BCTs employed are presented in Fig. 2. Of all 93 BCTs in the taxonomy, 19 (19/93, 22.9%) were used as active ingredients in the included interventions: four BCTs were used exclusively for interventions targeting health care consumers *(BCTs 3.3, 6.1, 9.2, 12.2)*; another four were used exclusively for multifaceted interventions that also targeted providers *(BCTs 1.3, 2.2, 3.2, 14.2)*, with 11 BCTs used for both (*BCTs 3.1, 4.1, 4.2, 5.1, 5.2, 8.2, 9.1, 10.1, 10.2, 12.1, 12.5;* see Tables 4 and 5 for details). When compared with the principles in the Behavioral Change Wheel, 39 interventions employed education as an active ingredient followed by enablement (*n* = 12), environmental restructuring (*n* = 8), and restriction (*n* = 4). Of the 43 included studies, 22 were interventions delivered only at the community level, 12 in primary care settings, six in both community and primary care settings, and three in schools. Nineteen interventions were delivered on an individual basis, which tended to be shorter in duration, ranging from one to multiple short sessions. The majority of studies focused on evaluation design and outcomes and only provided high-level descriptions of the intervention, with or without details on the development or implementation processes. Twenty studies provided clear descriptions on the intervention adaption/development process, all on implementation strategies (e.g., channels and timing of dissemination), and, to a certain level, 15 on intervention dose (intensity) [54–56] and nine on designs (e.g., color and format) [55–58]. Some studies provided links to intervention designs, but most of these links had expired. Only eight interventions explicitly reported having adopted a theory or model of behavioral change, which included social marketing [56, 59, 60], social cognitive theory [55], precede/proceed model [61], social development model [39, 40], and the health belief model [62]. However, little was reported on how these underlying theories were used in the development and evaluation of the interventions.

**Table 3:** Features of included interventions

First author, year	Gov’t support	Policy	Professional target						Public target															Multilingual
			Letters to doctors	Educational meetings (academic detailing)	Written materials	Clinical practice guidelines	Prescribing feedback	Physician financial incentives	TV	Video	Newsletters/mails	Poster	Radio	Press conferences	Newspapers or advertisements (including bill boards, bus signs)	Websites	Informational written materials (including pamphlets/brochures)	Education meetings	Mascots	School program (including peer-education)	Family and friends	Decision-aid/enabling tools	Other mass media campaign activities	
Belongia, 2001	Yes		X	X	X	X						X					X	X						NR
Belongia, 2005	Yes		X	X	X	X			X		X	X	X	X	X	X	X	X	X				X	Yes
Bernier, 2014	Yes		X	X	X	X			X		X	X	X	X	X	X	X	X					X	NR
Cebotarenco, 2008	No									X		X			X		X	X		X	X		X	NR
Finkelstein, 2001	Yes			X	X	X	X				X					X	X							NR
Finkelstein, 2008	Yes			X	X	X	X				X					X	X	X		X	X		X	NR
Formoso, 2013	Yes		X						X			X	X		X	X	X							NR
Fuertes, 2010	Yes								X							X								NR
Gonzales, 2004	Yes				X	X					X	X				X	X				X			Yes
Gonzales, 2005	Yes				X	X					X	X				X	X				X			Yes
Gonzales, 2008	Yes		X									X	X		X	X	X						X	Yes
Hennessy, 2002	Yes			X							X					X	X	X						NR
Kliemann, 2016	Yes	X																						NA
Lambert, 2007	Yes								X			X	X		X		X		X				X	NR
Lee, 2017	No																X	X						Yes
Mainous, 2009	No												X		X		X							Yes
McNuty, 2010	Yes		X		X	X						X			X		X							NR
Perz, 2002	Yes			X	X	X			X		X	X			X		X	X					X	NR
Sabuncu, 2009	Yes		X	X	X	X			X		X	X	X	X	X	X	X	X					X	NR
Santa-Ana-Tellez, 2013	Yes	X																						NA
Santa-Ana-Tellez, 2015	Yes	X																						NA
Taylor, 2005	Yes									X							X							NR
Trepka, 2001	Yes		X	X	X	X						X			X		X	X						NR
Wirtz, 2013	Yes	X																						NA
Wutzke, 2007	Yes		X	X	X		X		X			X	X	X	X	X	X	X					X	NR
Beshears, 2013	Yes										X													NR
O’Malley, 2006	No			X				X			X		X	X	X	X							X	NR
Sedjo, 2009	No			X							X						X							NR
Vallès, 2003	No																X	X						NR
Hasak, 2018	No															X	X							NR
Lawrence, 2019	No									X							X					X		NR
Maughan, 2016	No																X					X		NR
Rose, 2016	No																X							NR
Spoth, 2008	No															X				X	X			NR
Spoth, 2013	No															X				X	X			NR
Eden, 2014	No																X					X		Yes
Fraser, 1997	Yes																X	X			X			Yes
Hassani, 2016	No																	X						NR
Montgomery, 2007	No															X						X		NR
Navaee, 2015	No																X	X			X			NR
Sharifirad, 2013	No																X	X			X			NR
Shorten, 2005	No																X					X		NR
Valiani, 2014	No																X	X			X			NR

*NR* not reported

**Table 4:** Behavior change techniques and number of interventions targeting health care consumers and included specific behavior change techniques, behavior change techniques taxonomy volume 1 (BCTTv1) hierarchical clusters, and intervention content examples

BCT	BCTTv1 hierarchical clusters	Examples extracted from descriptions of the interventions	Frequency
*3.1 Social support (unspecified)*	**3. Social support**	*Educational programs for husbands of pregnant women that aimed to provide social support of husbands, which consequently reduces the rate of elective cesarean section.*	3
*3.3 Social support (emotional)*	**3. Social support**	*A resource person will provide peer influence during decision making process about mode of delivery*	1
*4.1 Instruction on how to perform the behavior*	**4. Shaping knowledge**	*Information about when antibiotics are and are not needed (e.g., rarely for bronchitis, not for colds).*	34
*4.2 Information about Antecedents*	**4. Shaping knowledge**	*Information about bacterial and viral infections*	22
*5.1 Information about health consequences*	**5. Natural consequences**	*Information about bacterial resistance or side effects of antibiotic use*	22
*5.2 Salience of consequences*	**5. Natural consequences**	*Emphasis on the consequences inappropriate use of antibiotics (e.g., antimicrobial resistance or side effects of antibiotic use)*	6
*6.1 Demonstration of the behavior*	**6. Comparison of behavior**	*Role play education to reduce the fear of childbirth*	3
*8.2 Behavior substitution*	**8. Repetition and substitution**	*Alternative remedies instead of antibiotics for colds*	11
*9.1 Credible source*	**9. Comparison of outcomes**	*Endorsement by CDC was designed to increase the credibility of key messages.*	4
*9.2 Pros and cons*	**9. Comparison of outcomes**	*Information about the differences between generic and brand-name drugs in terms of advantages (high-quality bioequivalent formulations, health professionals’ preferences, avoidance of confusions) and disadvantages (popularity, fidelity to branded products)*	8
*10.1 Material incentive (behavior)*	**10. Reward and threat**	*Switching to a lower-cost generic medication is cost-saving*	3
*10.2 Material reward (behavior)*	**10. Reward and threat**	*Associated cost savings to the recipient from switching to each of these alternatives*	3
*12.1 Restructuring the physical environment*	**12. Antecedents**	*Restriction on sale of antibiotics without prescription*	8
*12.2 Restructuring the social environment*	**12. Antecedents**	*Interventions focused on empirically supported family risk and protective factors, such as parental nurturing, child management skills, improved parent–adolescent communication skills and adolescent prosocial skill development (e.g., managing conflict and stress, handling peer pressure, developing positive friendships)*	3
*12.5 Adding objects to the environment*	**12. Antecedents**	*Mass media strategies were undertaken including advertising using billboards, television, radio, and magazines.*	12
15	**8**		143

**Table 5:** Behavior change techniques and number of interventions targeting health care providers that included specific behavior change techniques, behavior change techniques taxonomy volume 1 (BCTTv1) hierarchical clusters, and intervention content examples

BCT	BCTTv1 hierarchical clusters	Examples extracted from descriptions of the interventions	Frequency
*1.3 Goal setting (outcome)*	**1. Goals and planning**	*Provision of individual prescribing profiles depicting: (1) the proportion of adult bronchitis patients receiving antibiotic treatment (target 10 percent or less); (2) the proportion of these antibiotics belonging to a first-line group (erythromycin, doxycycline, tetracycline) (target 70% or more); and (3) the proportion of these antibiotics that are ineffective against proven bacterial causes of uncomplicated acute bronchitis (target 0%).*	1
*2.2 Feedback on behavior*	**2. Feedback and monitoring**	*Prescribing feedback, clinical audit with feedback*	3
*3.1 Social support (unspecified)*	**3. Social support**	*Interventions that inform best practice prescribing and that support health professionals manage patient expectations*	1
*3.2 Social support (practical)*	**3. Social support**	*This intervention will (1) provide a range of patient education materials to physician offices without charge, (2) provide ongoing information about antibiotic-use rates and resistance in the community, (3) provide feedback about prescribing by practice, and (4) serve as a general resource on issues of antibiotic prescribing and resistance*	3
*4.1 Instruction on how to perform the behavior*	**4. Shaping knowledge**	*Academic detailing to promote appropriate antibiotic use; practice guidelines which included with the patient profiles for adults with bronchitis and children with pharyngitis were compatible with those produced by the Centers for Disease Control and Prevention (CDC)*	15
*4.2 Information about Antecedents*	**4. Shaping knowledge**	*Clinical practice guidelines for common respiratory illnesses*	13
*5.1 Information about health consequences*	**5. Natural consequences**	*A reference card providing easy-to-read facts about symptoms and treatments for ARIs*	9
*5.2 Salience of consequences*	**5. Natural consequences**	*Emphasis on AMR*	2
*8.2 Behavior substitution*	**8. Repetition and substitution**	*Prescription pads with explanations on symptoms and appropriate treatment options (to be given to patients instead of antibiotic prescriptions)*	9
*9.1 Credible source*	**9. Comparison of outcomes**	*Endorsement by CDC was designed to increase the credibility of key messages.*	1
*10.1 Material incentive (behavior)*	**10. Reward and threat**	*An intervention intends to reward physicians for reducing pharmacy costs for their patients, one component of which was to increase their prescribing of generic drugs*	1
*10.2 Material reward (behavior)*	**10. Reward and threat**	*Reward given to physicians for reducing pharmacy costs for their patients, one component of which was to increase their prescribing of generic drugs*	1
*12.1 Restructuring the physical environment*	**12. Antecedents**	*Waiting room materials (CDC posters and patient reference cards)*	4
*12.5 Adding objects to the environment*	**12. Antecedents**	*Mass media strategies were undertaken including advertising using billboards, television, radio and magazines.*	10
*14.2 Punishment*	**14. Scheduled consequences**	*Regulations that require prescriptions for antibiotics to be retained and registered in pharmacies and imposes fines to the owners of the pharmacies for non-compliance.*	2
15	**10**		75

### Interventions targeting health care consumers

Table 4 reports the individual BCTs identified within the descriptions as active ingredients of the selected interventions targeting health care consumers. Of the 93 BCTs, the most frequently used active ingredients in the selected interventions targeting health care consumers were *BCTs: 4.1-Instruction on how to perform the behavior (n = 34), 4.2 Information about antecedents (n = 22)*, *5.1 Information about health consequences (n = 22),* followed by *12.5 Adding objects to the environment (n = 12), 8.2 Behavior substitution (n=11), and 12.1 Restructuring the physical environment (n = 8).* Most studies employed education interventions aiming to improve public knowledge (including awareness or correcting misconceptions). Mass media campaigns were widely used to reduce antibiotic misuse [54–56, 60, 63–68] and demand for brand-name drugs [69], all in HIC. The effectiveness of such behavioral change interventions was mixed. Decision aids to assist pregnant women making decisions about mode of delivery were tested in three different trials in Australia, UK, and USA; all reported to be ineffective [52, 70, 71]. Taylor et al. [72], Lee et al. [73], and Vallès et al. [51] trialed patient-based education interventions in primary care settings to reduce antibiotic use or to substitute generic for brand-name drugs; only Vallès et al.’s [51] intervention found a positive impact on behavior change. Mainous et al. and McNulty et al. assessed community-wide education interventions in the USA and UK on their effectiveness in improving public antibiotic use and found the provision of educational messages itself was insufficient to overcome the influence of past attitudes and behaviors [57, 66]. Formal and informal social support networks can be leveraged to influence individuals’ behaviors through improving doctor-patient communication [58–60, 64, 72, 74] or by actively engaging family members in the process [39, 40, 75]. Four interventions aimed to encourage disposal of leftover opioids among postoperative patients by employing a combination BCWs of education, enablement, and environment restructuring (BCTs: 4.1, 4.2, 5.1, 5.2, 8.2, 12.1, 12.5), which reported positive impact [76–79]. Two longitudinal RCTs on school-based universal preventive interventions in the USA that aimed to strengthen families and build life skills were introduced to middle schoolers [39, 40] and reported a lasting impact on preventing non-medical use of prescription drugs into adulthood. Structural environmental conditions regarding access to healthcare services and medicines, and promotive and restrictive policies—or the lack thereof—can be pathways to shaping individual behaviors. Two trend analyses assessing the effectiveness of French public education campaigns [63, 68] reported a significant reduction in antibiotic consumption rates; however, trials on community-wide public campaigns with academic detailing for practitioners did not demonstrate comparable levels of improvement in public antibiotic use. Belongia et al. and Fiskelstein et al. found little or no evidence—attributable to multi-year interventions in Wisconsin and Massachusetts—on reductions in antibiotic prescribing in the intervention areas, despite improved public knowledge [54, 59, 74]. Gonzales et al. found that the state-wide “Get Smart Colorado” campaign did not improve prescription rates, but might be associated with a reduction in antibiotic use in the community through decreases in office visit rates among children [56, 64]. Four studies evaluated the effectiveness of the restrictions on OCT purchases on antibiotic consumption in five Latin American countries with mixed results [33–35, 80].

### Interventions also targeting health care providers

Table 5 reports the individual BCTs identified within the descriptions as active ingredients of the selected interventions targeting health care providers. The most frequently used BCTs targeting health care providers were similar with those targeting consumers, with small differences in the ranking: *BCTs: 4.1 Instruction on how to perform the behavior (n = 15), 4.2 Information about antecedents (n = 13)*, *12.5 Adding objects to the environment (n = 10),* followed by *5.1 Information about health consequences (n = 9), 8.2 Behavior substitution (n = 9), and 12.1 Restructuring the physical environment (n = 4).* We noticed that, except for programs aiming to contain inappropriate use of antibiotics, other interventions had limited engagement between consumers and providers.

## Discussion

### Summary of findings

Using the Behavioral Change Wheel (BCW) domains to identify the theoretical concepts underlying interventions and the behavior change technique taxonomy v1 (BCTTv1) to identify the active ingredients of interventions, we found that the domain of education was the most commonly targeted by a majority of interventions with primary focus on the provision of information on *BCTs 4.1 how to perform the behavior* and *4.2 about antecedents and 5.1 the associated health consequences.* A plethora of evidence supports the view that human behaviors should be understood in their social ecological context, as products of intertwined influences at the personal, communal, societal, and structural levels [81–83]. Studies show that improving knowledge and awareness does not equate with appropriate behavior change, as lack of information is often not the only barrier to changing behavior [64, 66, 84–86]. The effects of education interventions have been mixed—most likely due to heterogeneity in context, population served, and intervention design and measures. Cabral et al. examined how communication affects prescription decisions for acute illnesses and demonstrated a clear miscommunication with cross-purposes between health care consumers and providers, as patients and/or caregivers focused on their concerns and information needs, which clinicians interpreted as an expectation for antibiotics [87]. This review supports the use of multifaceted (complex) interventions that incorporate BCTs related to provision of information (BCTs 4.1, 4.2, or 5.1) and, as an alternative to antibiotics, prescription pads with clear explanations on symptoms, and appropriate treatment options (BCT 8.2), as education alone is not sufficient to be effective. Interventions consisting of health education messages (e.g., BCTs 4.1, 4.2, 5.1), recommended behavior alternatives (BCT 8.2), and a supporting environment that incentivizes or encourages the adoption of a new behavior (e.g., BCTs 10.1, 10.2, 12.1, 12.5) are more likely to be successful. Other types of utilized behavior change techniques often aimed to encourage alternative behaviors and improve the physical environments via regulations or mass media.

The continuing tendency in research reporting has been to stress the effectiveness of interventions rather than the process of identifying and developing key components and the parameters within which they operate. There is a lack of detail on how the intervention components were selected, designed, and the process of implementing them, with limited descriptions provided on the “contexts” and “mechanisms” that determine the effectiveness of interventions. Few studies provided sufficient details on intervention development, dose/intensity, and design; some provided links to project materials that had expired [54–56, 60]. The majority of the selected interventions did not describe the pilot or process data for implementation, nor did they discuss the dissemination of findings and pathways to impact. Even after identifying active ingredients of interventions using BCTTv1, without a complete “recipe,” one cannot recreate successes in other contexts. Just like there are agreed-upon elements that constitute a rigorous and comprehensive reporting of evaluation studies, publications on behavioral change interventions should systematically cover a standardized list of intervention elements from the development, adaption and refinement, feasibility and pilot-testing, implementation, evaluation, and reporting of BCTs. The CONSORT-SPI team [88] has developed guidance and checklists for the reporting of BCT trials; however, the required details on the reporting are still primarily focused on evaluation study designs (e.g., process of randomization) rather than BCT development and implementation. From implementation research perspective and following the Medical Research Council (MRC) guidance on developing and evaluating complex interventions, reporting of BCT development and implementation should include descriptions on the context, target behavior determinants, theories and rationale (theory of change), intervention design features, adaption/development process, implementation strategy (e.g., implementor, dose/intensity), modifications made between the feasibility and effective assessment phases, and evaluation outcomes. The lack of detailed reporting among included intervention studies on evidence-based development and implementation processes undermines the generalizability of study findings, makes cross-intervention comparisons difficult, and complicates future adaption and replication efforts.

This systematic literature review is the first on the effectiveness of public-targeted behavioral change interventions to reduce inappropriate use of medical interventions. It identified a serious lack of formative data, which means that interventions to change public use of medical interventions are often designed on the basis of “best guesses” of what needs to change, without an evidence base or explicit rationale for the selection of a specific intervention strategy. There is an urgent need to adopt a multidisciplinary, systematic approach to developing evidence-based behavioral change interventions to reduce inappropriate medical use and to develop an operational mechanism for knowledge translation and scale-up within and across different countries. We found limited evidence [39, 63] on evaluating the impact of previous or ongoing education interventions on inappropriate use in terms of long-term impact, scalability, and replicability. The root causes of why certain interventions were unsuccessful are not systematically explored or reported, yet reporting “negative results” is likely as critical as reporting “active ingredients” and positive findings for the development and sustainability of implementation science.

### Relation to other studies

Like most stewardship programs, quaternary prevention—a relatively new category of medical prevention first raised in 1986 by Dr. Marc Jamoulle, a family physician, to addressing concerns around the protection of people and patients from being harmed by over-diagnosis or overtreatment—tends to focus mostly on health care providers while placing less attention on consumers [5, 89–91]. The definition of quaternary prevention was later expanded by Brodersen et al. in 2014 to include patients and medical interventions as an action taken to protect individuals (persons/patients) from medical interventions that are likely to cause more harm than good [92, 93]. The expanded definition recognizes the contemporary reality in medicine in which people may suffer harm from medical interventions throughout their entire lifetime—from conception to adulthood, in times of good health, as well as when experiencing self-limited disease, chronic conditions, or terminal disease. Therefore, quaternary prevention should include preventing all types of harm associated with medical interventions [92, 93]. From this perspective, quaternary prevention is aligned with the aims of the behavioral change interventions and techniques identified in our review and should be considered alongside the other four classical levels of preventive activities, i.e., primordial (e.g., laws that restrict over-the-counter purchases of antibiotics), primary (e.g., prescription drugs disposal programs), and secondary and tertiary preventions (e.g., interventions that reduce fear of childbirth or convert demand of brand-name drugs to generic drugs).

The use of medicine or medical procedures is a highly complex set of behaviors involving multiple actions, including the self-diagnostic process, assessing benefit/risk, decision-making around healthcare seeking and treatment choice, and review of treatment—each performed at different time points across the care continuum [94, 95]. It involves interactions with various stakeholders (i.e., family members and providers) and is often shaped more by individual and contextual factors than by a clinical diagnosis [94, 95]. Therefore, developers and implementers of behavioral change interventions should be clear as to whose and which behaviors are being targeted for change and how—namely, who needs to do what differently, how, to whom, where, when, and for how long. A set of precisely specified behaviors would allow for easier measurement and therefore would offer a baseline and metric for evaluating the success of an intervention.

In order to develop effective behavioral change interventions, we first need to explain why people behave in certain ways, yet a more in-depth look at people’s lifeworld is lacking from every reviewed article. As the dual processing theory (DPT) posits, human behavior is guided by two types of processing mechanisms: the implicit, intuitive system 1 and the explicit, rational system 2 [96]. Behavioral economists elaborate that, due to limited self-control, rationality and social preferences, actual decisions are less rational and stable than traditional normative theory suggests [96]. They are usually made with a range of biases resulting from the way people think and feel, rather than with rationality or full information. However, most of the included interventions—appealing to system 2 processing—attempted to influence behaviors via improved knowledge and attitudes; disappointingly, many trials indicated that this did not automatically lead to preferred behaviors [54, 59, 72, 74]. To complicate things further, Zinn argues that between rationality and irrationality, there is a third, “in-between” dimension that includes trust, intuition, and emotion, which is an important aspect of decision-making when people deal with risk and uncertainty, especially in anticipation of the possible undesired outcomes of decisions [97]. This may explain why three RCTs on decision aids (system 1) to address individual emotions (system 2) had no real impact on choice of vaginal birth [52, 70]. On the other hand, in addition to education programs, financial incentives (changes in co-payment), free medicine, advertisements (print media), and health policies have been experimented with as behavioral change interventions to influence healthcare consumers’ choice of medicine—in particular, to promote uptake of generic medicines—though they have demonstrated inconsistent results [98, 99].

The most promising measure was an intervention delivered face-to-face, where consumers were told that they had the option of switching back to brand-name drugs anytime [51, 100, 101]; hence, an intervention that leverages human behavioral mechanisms may be more effective and cost-effective in optimizing decision making than repeated, expensive education campaigns. In response to the recent opioid epidemic across the globe, promising prevention programs aimed not only to improve the knowledge and awareness of the risk of nonmedical use of prescription drugs among at risk individuals, but also to empower healthcare consumers by providing skills or tools that enable them to take action prior to the occurrence of misuse and/or before the development of poor habits [39, 40, 76–79]. These interventions further improved the socio-ecological surroundings of the target audience by involving family members and restructuring their social or physical environments [39, 40, 76–79].

Our review showed only 19% of BCTs have been utilized by included interventions (i.e., 81% of BCTs unexplored), with great variation between different types of misuse—most were limited to education. Future studies should explore other BCTs. A wide range of disciplines engaging in social and behavioral sciences, such as psychology, sociology, anthropology, communication, and marketing, can provide theories, models, and methods for a more comprehensive and coherent approach to understanding or even modifying contextual, organizational and interpersonal determinants of behavior. In terms of sustainability of the interventions themselves, other than a few longitudinal studies [39, 40], we do not know how long the reported effect of behavioral change will sustain. Few studies incorporated economic evaluations, and therefore, it was not possible to determine the returns on investment (ROI) for these included interventions. Future intervention studies should consider the aspects of RE-AIM *(Reach Effectiveness Adoption Implementation Maintenance)* framework or follow the MRC Guidelines on Developing and Evaluating Complex Interventions during the planning stage to enhance the impact of interventions and the reporting of them.

Development of a behavioral change intervention has to start with a realist, comprehensive understanding of the complex environment that shapes individual and collective behaviors. The etiology of inappropriate use of medical interventions should be studied and addressed within the context of its biological, psychosocial, behavioral, and environmental factors and the interactions between them. In early 2000, Sallies et al. developed a behavioral epidemiology framework, which specified a systematic sequence of studies on health-related behaviors leading to evidence-based interventions directed at populations in the following five phases: (1) establish links between behaviors and health, (2) develop measures of the behavior, (3) identify influences on the behavior, (4) evaluate interventions to change the behavior, and (5) translate research into practice [21, 83, 102]. In 2011, Michie and colleagues mapped out various pathways to influencing behavioral change and recommended that interventions seeking to change behavior should be designed on the basis of a thorough “behavioral diagnosis” of why behaviors are the way they are and what needs to change in order to bring about the desired behavior [21]. Conducting such diagnosis should be facilitated by the use of behavioral theory. Not until recent years did researchers systematically report efforts in the identification of the root causes of operational barriers and facilitators in designing, implementing, and evaluating interventions. For instance, in 2018 and 2019, Langdridge et al. have attempted to decipher the intervention elements and visual imagery used in public antimicrobial stewardship [23, 103].

Consistent with the findings from recent reviews by Cochrane and the Department of Health and Social Care and Public Health in England [5, 104, 105], our review found that few interventions employed behavior change theories or techniques. Behavioral determinants and social influences are often not given sufficient consideration in the design and evaluations of interventions. To inform the design of effective, context-specific behavior change interventions, one must first define the problem in both behavioral terms and in its current context and adopt a theory-driven, systematic approach to intervention design. This points to another critical knowledge gap identified by this review in implementation science, namely early studies that take place prior to the implementation of behavioral change interventions. Following the Medical Research Council (MRC) guidelines on developing and evaluating complex interventions [106], as presented in Table 1, we find there is little reporting on the feasibility, pilot, or process data that generates the needed contextual information and evidence base for acceptance, adaption, and uptake. Limited detail has been made available on the development of the included interventions regarding how key decisions were made, including feasibility and compliance. Future research on pilot and/or feasibility studies that aim to strengthen large-scale behavioral change intervention design can span the continuum of implementation science research from idea generation to intervention development, implementation, evaluation, and scale-up.

### Limitations

This systematic review is subject to important limitations as we worked with interventions that are complex, heterogeneous, non-standardised, and targeted different types of inappropriate use of medical interventions and users. The diversity in the design and outcome measures of the included interventions prevents us from performing a meta-analysis. We demonstrated great variability in the effect size observed within each behavioral change intervention considered. We cannot make a conclusion that certain types of behavioral change intervention might be more effective than any other type of design due to the limitations of the literature relating to the lack of evidence-based development process and evaluation design. Behavioral data that were gathered via survey instruments were by nature self-reported from health care consumers who may have been reluctant to report practices that could be considered inappropriate or may have been subject to recall bias. Often there were more than one “active ingredient” identified for each included intervention, yet retrospective coding and the study design did not allow us to pinpoint which component was more effective. Further, some studies contained bundles of interventions while others contained similar, yet different interventions implemented in multiple countries; therefore, the results of this review may have been clouded by unconsidered/unreported intervention components in the studies included. The studies in this review were spread across a wide range of settings and populations, so general conclusions should be drawn with caution. Publication bias may be a critical problem since it implies that most interventions have a positive effect. We expect most interventions aimed at individuals to be much more complex in reality; however, this review was not able to capture how and why “active ingredients” were selected, implemented, or functioned in the respective socioeconomical, cultural, and healthcare settings. Future work should focus on addressing the limitations and uncertainties surrounding existing behavioral change interventions.

## Conclusion

Systematically assessing the evidence across behavioral change interventions allows for the identification of the “active ingredients” of effective interventions that improve healthcare consumers’ use of medical interventions, as well as the identification of those with ineffective or uncertain outcomes. Although opportunities for behavioral change interventions are becoming more commonly recognized, multifaceted (complex) interventions are still new, scarce, limited to high-income countries, and, as is evident from our findings, highly heterogeneous. Public-targeted behavioral change interventions in low- and middle-income countries (LMICs) were exclusively limited to primary care settings. Interventions that consist of health education messages, recommended behavior alternatives, and a supporting environment that incentivizes or encourages the adoption of a new behavior are more likely to be successful. Future research should also seek to unpack the distinctions between various audience segments, the influence of the social ecological context, and the utility of the unexplored 81% of behavioral change techniques (BCTs). It is critical to adhere to a rigorous framework that guides the development, implementation, evaluation, and reporting of evidence-based interventions, so that generated evidence can be documented, disseminated, compared, and utilized for further research. The lack of reporting on evidence-based development and implementation processes makes cross-intervention comparisons and replication difficult. Our review further identified a need for standardized reporting of intervention development, adaptation, and implementation to maximize generalisability and replicability.

## Supplementary information

**Additional file 1:.** Search Strategy

**Additional file 2:.** Inclusion and Exclusion Criteria

**Additional file 3:.** List of included studies

**Additional file 4:.** Summary of quality assessment of included studies

## Data Availability

Not applicable.

## Abbreviations

ABR: Antibiotic resistance
AMR: Antimicrobial resistance
BCT: Behavior change technique
BCTT: Behavior change technique taxonomy
BCW: Behavioral Change Wheel
CPP: Controlled pre- and post-study
CRT: Cluster randomized control trial
CS: Elective cesarean section
DPT: Dual processing theory
EPHPP: Effective Public Health Practice Project’s Quality Assessment Tool for Quantitative Studies
HIC: High-income country
ITS: Interrupted time series
LMIC: Low- and middle-income country
MoD: Mode of delivery
MRC: Medical research council
NA: Not applicable
NR: Not reported
NCT: Nonrandomized controlled trial
OTC: Over-the-counter purchases
PDM: Prescription drug misuse
PRISMA: Preferred Reporting Items for Systematic Reviews and Meta-Analyses
RCT: Randomized control trial
ROI: Returns on investment
VD: Normal vaginal delivery
WHO: World Health Organization
