# Supplementary material for: Public target interventions to reduce the inappropriate use of medicines or medical procedures: a systematic review
Source: Implement Sci. 2020 Oct 20;15:90. doi: 10.1186/s13012-020-01018-7 (PMC7574316; doi:10.1186/s13012-020-01018-7)
Supplement: Supplementary file 1 — Additional file 1:. Search Strategy [file 13012_2020_1018_MOESM1_ESM.docx]

**Additional file 1. Search Strategy**

| **Database** | **Search Strategy** | **Results** |
| --- | --- | --- |
| PubMed | (((((((((AMR[tiab] OR antimicrobial resistance[tiab] OR antimicrobial[tiab] OR antibiotic*[tiab] OR caesarean Section*[mesh] OR C section[tiab] OR Caesarean[tiab] OR topical corticosteroid OR prescription drug* OR Drug Utilization[Mesh] OR generic drugs[Mesh] OR Anti-Bacterial Agents/therapeutic use*[Mesh] OR Opiate[tiab] OR opioid[tiab]))) AND ((behavior and behavior mechanisms[Mesh] OR choice behavior[Mesh] OR health knowledge, attitudes, practice*[Mesh] OR usage[tiab] OR use[tiab] OR consum*[tiab] OR behavior*[tiab] OR behavior*[tiab]))) AND ((education[tiab] OR campaign*[tiab] OR patient education as topic/methods[Mesh] OR health communication[Mesh] OR health education[Mesh] OR health promotion/utilization*[Mesh] OR social media[Mesh] OR communication[Mesh] OR communication[tiab] OR intervention*[tiab] OR strateg*[tiab] OR program*[tiab] OR media[tiab] OR mass media[Mesh] OR initiat*[tiab]))) AND ((((evidence-based Practice*[Mesh] OR Epidemiologic Methods[Mesh] OR evaluat*[tiab] OR assess*[tiab] OR effect*[tiab] OR empirical*[tiab] OR evidence[tiab] OR Evaluation Studies as Topic[Mesh] OR Program Evaluation*[Mesh] OR Evaluation Studies[pt] OR Randomized Controlled Trial[pt]) OR impact[tiab]))))) NOT (((((((((((animals[MeSH Terms]) OR depression[MeSH Terms]) OR economics[MeSH Terms]) OR intensive care units[MeSH Terms]) OR practice guidelines as topic[MeSH Terms]) OR inpatients[MeSH Terms]) OR mental disorders[MeSH Terms]) OR bacterial genome[MeSH Terms]) OR ((surge*[Title/Abstract] OR addiction[Title/Abstract] OR inject*[Title/Abstract])))) | 1378 |
| EMBASE | ('evidence-based'/exp OR 'evidence-based' OR 'evidence'/exp OR 'evidence' OR 'empirical' OR 'evaluat*':ab,ti OR 'assess*':ab,ti OR 'effect*':ab,ti) AND ('health education'/exp OR 'interpersonal communication'/exp OR 'intervention study'/exp OR 'behavior'/exp OR 'awareness'/exp OR 'health promotion'/exp OR 'patient education'/exp OR 'social media'/exp OR 'attitude to health'/exp OR 'health communication'/exp OR 'campaign*':ab,ti OR 'strateg*':ab,ti) AND (('misuse':ab,ti OR 'overuse':ab,ti OR 'drug abuse':ab,ti) AND ('antibiotic agent'/exp OR 'antibiotic*':ab,ti OR 'opioid':ab,ti OR 'caesarean section':ab,ti OR 'topical corticosteroid':ab,ti OR 'prescription drug'/exp OR 'drug utilization'/exp) OR 'generic drug':ab,ti) NOT [animals]/lim NOT [medline]/lim AND [1-1-1900]/sd NOT [1-6-2019]/sd | 1110 |
| PsycINFO | ( ((((((MA evidence-based Practice* OR MA Epidemiologic Methods OR AB evaluat* OR AB assess* OR AB effect* OR AB empirical* OR AB evidence))) AND ((AB education OR AB campaign* OR MA patient education as topic/methods OR MA health communication OR MA health education OR health policy OR MA health promotion/ utilization* OR MA social media/ utilization OR MA communication OR AB communication OR intervention* OR strateg* OR program* OR MA access to information OR AB media OR MA mass media OR AB initiat*))) AND ((MA behavior and behavior mechanisms OR MA choice behavior OR MA health knowledge, attitudes, practice* OR AB usage OR AB use OR AB consum* OR AB behavior* OR AB behavior* OR AB "practice*"))) AND ((misuse OR overuse) AND (AB AMR OR AB antimicrobial resistance OR AB antibiotic*) OR MA caesarean Section* OR AB C section OR AB Caesarean OR AB topical corticosteroid OR AB prescription drug* OR MA Drug Utilization OR MA generic drugs OR MA anti-bacterial agents/therapeutic use* OR AB Opiate OR AB opioid))) NOT ((animal* OR AB surgery OR AB Surgical OR AB dental OR AB cancer* OR AB Chronic obstructive pulmonary disease OR AB COPD OR AB alcohol OR AB tobacco OR AB addiction OR AB depression OR AB disorder* OR AB adherence OR AB diabet* OR MA Inpatients* OR AB inpatient* OR MA Hospitals OR AB tertiary OR AB HIV OR AB tuberculosis OR MA Practice Guidelines as Topic OR emergency[ti] OR ED[tiab] OR MA Intensive Care Units OR MA Practice Patterns, Physicians’ OR MA Economics OR AB steward* OR AB analgesic* OR MA Hospitalization OR MA Health Care Facilities OR MA Health Care Facilities OR MA Patient Care Management)) NOT PO animal ) NOT Direct-to-consumer NOT AB inject | 1557 |
