# Supplementary material for: Public target interventions to reduce the inappropriate use of medicines or medical procedures: a systematic review
Source: Implement Sci. 2020 Oct 20;15:90. doi: 10.1186/s13012-020-01018-7 (PMC7574316; doi:10.1186/s13012-020-01018-7)
Supplement: Supplementary file 2 — Additional file 2:. Inclusion and Exclusion Criteria [file 13012_2020_1018_MOESM2_ESM.docx]

**Additional file 2. Inclusion and Exclusion Criteria**

|  | Inclusion criteria | Exclusion criteria |
| --- | --- | --- |
| Type of inappropriate or unnecessary use of medical services or medicine | antibiotic use  elective caesarean section  nonmedical use of prescription drugs  demand for brand-named drugs  other types of inappropriate or unnecessary use of medical services or medicine | interventions based solely in clinical settings and relying on clinicians’ participation |
| Language | All | none |
| Time period | inception of databases to May 2019 | none |
| Population | general public   - children (age < 18 years) - pregnant women | - clinicians and other healthcare staff - animal |
| Intervention | Non-clinical interventions that aim to change behaviors for the reduction of inappropriate medical services or medicine use on demand side, and were assessed with robust evaluation data | interventions that pertain to:  behaviors of clinician, pharmacists, or prescribers  treatments for impatient  treatments for emergency services  clinical guidelines  stewardship programs targeting clinicians or providers   - dental setting - cancer treatment - addiction - mental health - tuberculosis - clinical treatment - HIV treatment   direct-to-consumer advertisement   - alcohol or tobacco use - substance abuse |
| Outcome | Reduction in: antibiotic use, the public’s antibiotic-related behavior, or other types of inappropriate/unnecessary medical services or medicine use | outcomes that were not changes in consumption or behaviors  outcomes that mainly focused on knowledge or attitudes, but not on behaviors. |
| Study Design | Randomized controlled trial (RCT)  Cluster randomized controlled trial (CRT)  Nonrandomised controlled trial (NCT)  quasi-experiments: interrupted time series (with at least three data points before and three after the intervention) and  controlled before-and-after studies | - editorials or commentaries - modelling - study protocols - reviews or literature reviews - descriptive studies - observational studies without evaluation data - studies reported evaluation data but did not employ a control group and/or report baseline data - Time series analysis that do not have a clearly defined point in time when the intervention occurred and at least three - data points before and three after the intervention - cost analysis or cost-effective analysis without behavioral data   economy evaluation |
