# Supplementary material for: Public target interventions to reduce the inappropriate use of medicines or medical procedures: a systematic review
Source: Implement Sci. 2020 Oct 20;15:90. doi: 10.1186/s13012-020-01018-7 (PMC7574316; doi:10.1186/s13012-020-01018-7)
Supplement: Supplementary file 3 — Additional file 3:. List of included studies [file 13012_2020_1018_MOESM3_ESM.docx]

**Additional file 3. List of included studies**

1. Belongia EA, Knobloch MJ, Kieke BA, Davis JP, Janette C, Besser RE. Impact of statewide program to promote appropriate antimicrobial drug use. *Emerg Infect Dis* 2005; **11**(6): 912-20.

2. Belongia EA, Sullivan BJ, Chyou PH, Madagame E, Reed KD, Schwartz B. A community intervention trial to promote judicious antibiotic use and reduce penicillin-resistant Streptococcus pneumoniae carriage in children. *Pediatrics* 2001; **108**(3): 575-83.

3. Bernier A, Delarocque-Astagneau E, Ligier C, Vibet MA, Guillemot D, Watier L. Outpatient antibiotic use in France between 2000 and 2010: after the nationwide campaign, it is time to focus on the elderly. *Antimicrobial agents and chemotherapy* 2014; **58**(1): 71-7.

4. Beshears J, Choi JJ, Laibson D, Madrian BC, Reynolds G. Testimonials do not convert patients from brand to generic medication. *The American journal of managed care* 2013; **19**(9): e314-31.

5. Cebotarenco N, Bush PJ. Reducing antibiotics for colds and flu: a student-taught program. *Health Educ Res* 2008; **23**(1): 146-57.

6. Eden KB, Perrin NA, Vesco KK, Guise JM. A randomized comparative trial of two decision tools for pregnant women with prior cesareans. *Journal of obstetric, gynecologic, and neonatal nursing : JOGNN* 2014; **43**(5): 568-79.

7. Finkelstein JA, Davis RL, Dowell SF, et al. Reducing antibiotic use in children: a randomized trial in 12 practices. *Pediatrics* 2001; **108**(1): 1-7.

8. Finkelstein JA, Huang SS, Kleinman K, et al. Impact of a 16-community trial to promote judicious antibiotic use in Massachusetts. *Pediatrics* 2008; **121**(1): e15-23.

9. Formoso G, Paltrinieri B, Marata AM, et al. Feasibility and effectiveness of a low cost campaign on antibiotic prescribing in Italy: community level, controlled, non-randomised trial. *Bmj* 2013; **347**: f5391.

10. Fraser W, Maunsell E, Hodnett E, Moutquin JM. Randomized controlled trial of a prenatal vaginal birth after cesarean section education and support program. Childbirth Alternatives Post-Cesarean Study Group. *American journal of obstetrics and gynecology* 1997; **176**(2): 419-25.

11. Fuertes EI, Henry B, Marra F, Wong H, Patrick DM. Trends in antibiotic utilization in Vancouver associated with a community education program on antibiotic use. *Canadian journal of public health = Revue canadienne de sante publique* 2010; **101**(4): 304-8.

12. Gonzales R, Corbett KK, Leeman-Castillo BA, et al. The "minimizing antibiotic resistance in Colorado" project: impact of patient education in improving antibiotic use in private office practices. *Health services research* 2005; **40**(1): 101-16.

13. Gonzales R, Corbett KK, Wong S, et al. "Get smart Colorado": impact of a mass media campaign to improve community antibiotic use. *Medical care* 2008; **46**(6): 597-605.

14. Gonzales R, Sauaia A, Corbett KK, et al. Antibiotic treatment of acute respiratory tract infections in the elderly: effect of a multidimensional educational intervention. *Journal of the American Geriatrics Society* 2004; **52**(1): 39-45.

15. Hasak JM, Roth Bettlach CL, Santosa KB, Larson EL, Stroud J, Mackinnon SE. Empowering Post-Surgical Patients to Improve Opioid Disposal: A Before and After Quality Improvement Study. *J Am Coll Surg* 2018; **226**(3): 235-40.e3.

16. Hassani L, Aghamolaei T, Ghanbarnejad A, Dadipoor S. The effect of an instructional program based on health belief model in decreasing cesarean rate among primiparous pregnant mothers. *J Educ Health Promot* 2016; **5**: 1.

17. Hennessy TW, Petersen KM, Bruden D, et al. Changes in antibiotic-prescribing practices and carriage of penicillin-resistant Streptococcus pneumoniae: A controlled intervention trial in rural Alaska. *Clin Infect Dis* 2002; **34**(12): 1543-50.

18. Kliemann BS, Levin AS, Moura ML, Boszczowski I, Lewis JJ. Socioeconomic Determinants of Antibiotic Consumption in the State of Sao Paulo, Brazil: The Effect of Restricting Over-The-Counter Sales. *PLoS One* 2016; **11**(12): e0167885.

19. Lambert MF, Masters GA, Brent SL. Can mass media campaigns change antimicrobial prescribing? A regional evaluation study. *The Journal of antimicrobial chemotherapy* 2007; **59**(3): 537-43.

20. Lawrence AE, Carsel AJ, Leonhart KL, et al. Effect of Drug Disposal Bag Provision on Proper Disposal of Unused Opioids by Families of Pediatric Surgical Patients: A Randomized Clinical Trial. *JAMA pediatrics* 2019: e191695.

21. Lee MHM, Pan DST, Huang JH, et al. Results from a Patient-Based Health Education Intervention in Reducing Antibiotic Use for Acute Upper Respiratory Tract Infections in the Private Sector Primary Care Setting in Singapore. *Antimicrobial agents and chemotherapy* 2017; **61**(5).

22. Mainous AG, 3rd, Diaz VA, Carnemolla M. A community intervention to decrease antibiotics used for self-medication among Latino adults. *Annals of family medicine* 2009; **7**(6): 520-6.

23. Maughan BC, Hersh EV, Shofer FS, et al. Unused opioid analgesics and drug disposal following outpatient dental surgery: A randomized controlled trial. *Drug and alcohol dependence* 2016; **168**: 328-34.

24. McNulty CA, Nichols T, Boyle PJ, Woodhead M, Davey P. The English antibiotic awareness campaigns: did they change the public's knowledge of and attitudes to antibiotic use? *The Journal of antimicrobial chemotherapy* 2010; **65**(7): 1526-33.

25. Montgomery AA, Emmett CL, Fahey T, et al. Two decision aids for mode of delivery among women with previous caesarean section: randomised controlled trial. *Bmj* 2007; **334**(7607): 1305.

26. Navaee M, Abedian Z. Effect of role play education on primiparous women's fear of natural delivery and their decision on the mode of delivery. *Iranian journal of nursing and midwifery research* 2015; **20**(1): 40-6.

27. O'Malley AJ, Frank RG, Kaddis A, Rothenberg BM, McNeil BJ. Impact of alternative interventions on changes in generic dispensing rates. *Health services research* 2006; **41**(5): 1876-94.

28. Perz JF, Craig AS, Coffey CS, et al. Changes in antibiotic prescribing for children after a community-wide campaign. *Jama* 2002; **287**(23): 3103-9.

29. Rose P, Sakai J, Argue R, Froehlich K, Tang R. Opioid information pamphlet increases postoperative opioid disposal rates: a before versus after quality improvement study. *Canadian journal of anaesthesia = Journal canadien d'anesthesie* 2016; **63**(1): 31-7.

30. Sabuncu E, David J, Bernede-Bauduin C, et al. Significant reduction of antibiotic use in the community after a nationwide campaign in France, 2002-2007. *PLoS Med* 2009; **6**(6): e1000084.

31. Santa-Ana-Tellez Y, Mantel-Teeuwisse AK, Dreser A, Leufkens HG, Wirtz VJ. Impact of over-the-counter restrictions on antibiotic consumption in Brazil and Mexico. *PLoS One* 2013; **8**(10): e75550.

32. Santa-Ana-Tellez Y, Mantel-Teeuwisse AK, Leufkens HG, Wirtz VJ. Seasonal variation in penicillin use in Mexico and Brazil: analysis of the impact of over-the-counter restrictions. *Antimicrobial agents and chemotherapy* 2015; **59**(1): 105-10.

33. Sedjo RL, Cox ER. The influence of targeted education on medication persistence and generic substitution among consumer-directed health care enrollees. *Health services research* 2009; **44**(6): 2079-92.

34. Sharifirad G, Rezaeian M, Soltani R, Javaheri S, Mazaheri MA. A survey on the effects of husbands' education of pregnant women on knowledge, attitude, and reducing elective cesarean section. *J Educ Health Promot* 2013; **2**: 50.

35. Shorten A, Shorten B, Keogh J, West S, Morris J. Making choices for childbirth: a randomized controlled trial of a decision-aid for informed birth after cesarean. *Birth (Berkeley, Calif)* 2005; **32**(4): 252-61.

36. Spoth R, Trudeau L, Shin C, et al. Longitudinal effects of universal preventive intervention on prescription drug misuse: three randomized controlled trials with late adolescents and young adults. *Am J Public Health* 2013; **103**(4): 665-72.

37. Spoth R, Trudeau L, Shin C, Redmond C. Long-term effects of universal preventive interventions on prescription drug misuse. *Addiction (Abingdon, England)* 2008; **103**(7): 1160-8.

38. Taylor JA, Kwan-Gett TS, McMahon EM, Jr. Effectiveness of a parental educational intervention in reducing antibiotic use in children: a randomized controlled trial. *The Pediatric infectious disease journal* 2005; **24**(6): 489-93.

39. Trepka MJ, Belongia EA, Chyou PH, Davis JP, Schwartz B. The effect of a community intervention trial on parental knowledge and awareness of antibiotic resistance and appropriate antibiotic use in children. *Pediatrics* 2001; **107**(1): E6.

40. Valiani M, Haghighatdana Z, Ehsanpour S. Comparison of childbirth training workshop effects on knowledge, attitude, and delivery method between mothers and couples groups referring to Isfahan health centers in Iran. *Iranian journal of nursing and midwifery research* 2014; **19**(6): 653-8.

41. Valles JA, Barreiro M, Cereza G, et al. A prospective multicenter study of the effect of patient education on acceptability of generic prescribing in general practice. *Health policy (Amsterdam, Netherlands)* 2003; **65**(3): 269-75.

42. Wirtz VJ, Herrera-Patino JJ, Santa-Ana-Tellez Y, Dreser A, Elseviers M, Vander Stichele RH. Analysing policy interventions to prohibit over-the-counter antibiotic sales in four Latin American countries. *Trop Med Int Health* 2013; **18**(6): 665-73.

43. Wutzke SE, Artist MA, Kehoe LA, Fletcher M, Mackson JM, Weekes LM. Evaluation of a national programme to reduce inappropriate use of antibiotics for upper respiratory tract infections: effects on consumer awareness, beliefs, attitudes and behaviour in Australia. *Health promotion international* 2007; **22**(1): 53-64.
