# Supplementary material for: Public target interventions to reduce the inappropriate use of medicines or medical procedures: a systematic review
Source: Implement Sci. 2020 Oct 20;15:90. doi: 10.1186/s13012-020-01018-7 (PMC7574316; doi:10.1186/s13012-020-01018-7)
Supplement: Supplementary file 4 — Additional file 4:. Summary of quality assessment of included studies [file 13012_2020_1018_MOESM4_ESM.docx]

**Additional file 4. Summary of quality assessment of included studies**

|  | Selection Bias | Study Design | Confounders | Blinding | Data Collection Methods | Withdrawal and Drop-outs | Overall Rating |
| --- | --- | --- | --- | --- | --- | --- | --- |
| Belongia, 2001 | Weak | Moderate | Moderate | Weak | Strong | Weak | WEAK |
| Belongia, 2005 | Weak | Moderate | Moderate | Moderate | Strong | Strong | MODERATE |
| Bernier, 2014 | Strong | Moderate | Moderate | Moderate | Moderate | Moderate | STRONG |
| Cebotarenco, 2008 | Moderate | Weak | Moderate | Weak | Weak | Weak | WEAK |
| Finkelstein, 2001 | Moderate | Strong | Strong | Moderate | Strong | Strong | STRONG |
| Finkelstein, 2008 | Moderate | Strong | Strong | Moderate | Strong | Strong | STRONG |
| Formoso, 2013 | Moderate | Strong | Moderate | Moderate | Strong | Strong | STRONG |
| Fuertes, 2010 | Strong | Moderate | Moderate | Moderate | Moderate | Moderate | STRONG |
| Gonzales, 2004 | Weak | Moderate | Moderate | Moderate | Strong | Strong | MODERATE |
| Gonzales, 2005 | Weak | Moderate | Moderate | Moderate | Strong | Strong | MODERATE |
| Gonzales, 2008 | Moderate | Strong | Strong | Moderate | Strong | Strong | STRONG |
| Hennessy, 2002 | Moderate | Strong | Weak | Moderate | Strong | Strong | MODERATE |
| Kliemann, 2016 | Strong | Moderate | Weak | Moderate | Strong | Strong | MODERATE |
| Lambert, 2007 | Moderate | Weak | Weak | Moderate | Moderate | Weak | WEAK |
| Lee, 2017 | Weak | Strong | Weak | Weak | Moderate | Strong | WEAK |
| Mainous, 2009 | Weak | Weak | Moderate | Weak | Weak | Weak | WEAK |
| McNulty, 2010 | Moderate | Weak | Weak | Weak | Weak | Moderate | WEAK |
| Perz, 2002 | Moderate | Moderate | Weak | Moderate | Strong | Strong | MODERATE |
| Sabuncu, 2009 | Strong | Moderate | Moderate | Moderate | Moderate | Moderate | STRONG |
| Santa-Ana-Tellez, 2013 | Strong | Moderate | Moderate | Moderate | Moderate | Moderate | STRONG |
| Santa-Ana-Tellez, 2015 | Strong | Moderate | Moderate | Moderate | Moderate | Moderate | STRONG |
| Taylor, 2005 | Weak | Weak | Weak | Moderate | Strong | Strong | WEAK |
| Trepka, 2001 | Weak | Moderate | Moderate | Weak | Weak | Weak | WEAK |
| Wirtz, 2013 | Strong | Moderate | Weak | Moderate | Strong | Strong | MODERATE |
| Wutzke, 2007 | Strong | Moderate | Weak | Moderate | Moderate | Moderate | MODERATE |
| Beshears, 2013 | Moderate | Moderate | Weak | Moderate | Strong | Strong | MODERATE |
| O'Malley, 2006 | Moderate | Weak | Strong | Moderate | Strong | Strong | MODERATE |
| Sedjo, 2009 | Moderate | Moderate | Weak | Moderate | Strong | Strong | MODERATE |
| Vallès, 2003 | Moderate | Moderate | Strong | Moderate | Strong | Strong | STRONG |
| Hasak 2018 | Weak | Weak | Weak | Weak | Weak | Moderate | WEAK |
| Lawrence, 2019 | Moderate | Strong | Moderate | Weak | Moderate | Strong | MODERATE |
| Maughan, 2016 | Weak | Moderate | Weak | Weak | Weak | Strong | WEAK |
| Rose, 2016 | Weak | Weak | Weak | Weak | Weak | Moderate | WEAK |
| Spoth, 2008 | Moderate | Moderate | Moderate | Weak | Moderate | Weak | WEAK |
| Spoth, 2013 | Moderate | Moderate | Moderate | Weak | Moderate | Weak | WEAK |
| Eden, 2014 | Weak | Moderate | Weak | Moderate | Strong | Moderate | WEAK |
| Fraser, 1997 | Weak | Moderate | Weak | Moderate | Strong | Strong | WEAK |
| Hassani, 2016 | Weak | Weak | Weak | Weak | Moderate | Strong | WEAK |
| Montgomery, 2007 | Moderate | Moderate | Moderate | Moderate | Strong | Strong | STRONG |
| Navaee, 2015 | Weak | Moderate | Weak | Moderate | Weak | Strong | WEAK |
| Sharifirad, 2013 | Weak | Moderate | Weak | Weak | Weak | Strong | WEAK |
| Shorten, 2005 | Weak | Moderate | Weak | Moderate | Strong | Moderate | WEAK |
| Valiani, 2014 | Weak | Moderate | Weak | Weak | Weak | Strong | WEAK |
